# Understanding the global rise of artemisinin resistance: Insights from over 100,000 *Plasmodium falciparum* samples

**DOI:** 10.7554/eLife.105544

**Published:** 2025-10-02

**Authors:** Andrew J Balmer, Nina FD White, Eyyüb S Ünlü, Chiyun Lee, Richard D Pearson, Jacob Almagro-Garcia, Cristina Ariani

**Affiliations:** 1 Genomic Surveillance Unit, Wellcome Sanger Institute Hinxton United Kingdom; University of São Paulo Brazil; University of the Witwatersrand South Africa

**Keywords:** artemisinin, resistance, *Plasmodium falciparum*, genomic surveillance, artemisinin combination therapy, malaria, *Plasmodium falciparum*

## Abstract

Artemisinin partial resistance (ART-R) in *Plasmodium falciparum* is a major challenge to malaria control globally. Over the last two decades, ART-R has spread widely across Southeast Asia, undermining public health strategies and hindering elimination. As of 2024, ART-R has now emerged in East Africa, with the potential to dramatically impact current efforts to control malaria in the region. Mitigating its spread requires detailed genomic surveillance of point mutations in the *kelch13* gene, the primary known determinant of artemisinin resistance. Although extensive surveillance data on these markers is available, it is distributed across many literature studies and open databases. In this review, we aggregate spatiotemporal data for 112,933 *P. falciparum* samples collected between 1980 and 2023 into a single resource, providing the most comprehensive overview of *kelch13* markers to date. We outline the history and current status of these mutations globally, with particular focus on their emergence in Southeast Asia and East/Northeast Africa. Concerningly, we find the recent increases in ART-R in Africa mirror patterns observed in Southeast Asia 10–15 years ago. We examine factors that may influence its spread, including fitness costs, treatment strategies, and local epidemiological dynamics, before discussing potential scenarios for how resistance may spread in Africa in coming years. This review provides a comprehensive account of how the situation of ART-R has unfolded globally so far, highlighting insights for researchers and public health bodies which aim to reduce its negative effects.

## Introduction

Artemisinin-based combination therapies (ACTs) are the most important antimalarial treatment currently available. By combining a fast-acting artemisinin derivative with a longer-acting partner drug, ACTs provide a highly effective treatment for uncomplicated malaria. The artemisinin derivative reduces parasite biomass over the first 3 days of treatment, before the partner drug clears the remaining infection (Nosten and White, 2007). Because of their effectiveness, the WHO has recommended ACTs as primary treatment for uncomplicated malaria, and as a follow-up treatment for severe malaria, since 2006 (World Health Organization, 2006). As of 2024, ACTs remain the most widely used antimalarial globally in treating infection by *Plasmodium falciparum* and have been adopted as a primary malaria treatment strategy by 84 countries (World Health Organization, 2022b). Currently, around 65% of all uncomplicated malaria cases are treated with ACTs, with up to 70% of infections in Africa treated using the ACT artemether-lumefantrine (Dhorda et al., 2024). While new drugs are in development to replace ACTs, the lengthy testing and clinical trial process means ACTs will likely remain the primary treatment for the foreseeable future (Erhunse and Sahal, 2021).

Given their critical role in malaria treatment, preventing resistance to ACTs remains a major priority for the malaria community. Initially, there was optimism that resistance to artemisinin may not occur, or would occur more slowly than resistance to previous drugs, due to the combination of an artemisinin derivative with a partner drug (Yeung et al., 2004; Pongtavornpinyo et al., 2008). However, this optimism faded in 2008, when reports of reduced efficacy of artemisinin-based drugs emerged from the Thai-Cambodian border. These were the first recorded instances of what we now refer to as artemisinin ‘partial resistance’ (ART-R) (Noedl et al., 2008; Dondorp et al., 2009; Ashley et al., 2014; World Health Organization, 2016). ART-R is characterised by delayed parasite clearance time following treatment with an artemisinin derivative, where high parasitaemia persists beyond day 3 of treatment, or if parasite clearance half-life exceeds 5 hours (Rosenthal, 2021). It is termed ‘partial’ resistance because parasite clearance is delayed rather than prevented, leaving more parasites for the partner drug to clear (Behrens et al., 2021). While this does not immediately lead to treatment failure, it increases selective pressure for partner drug resistance, potentially leading to parasites which are resistant to both drugs and a higher likelihood of overall treatment failure.

After emerging on the Thai-Cambodia border in 2008, ART-R then spread across the Greater Mekong Subregion into Myanmar, Vietnam, and Laos throughout the early 2010s (Ashley et al., 2014; Tun et al., 2015). As with resistance to previous antimalarials chloroquine and sulfadoxine–pyrimethamine (Roux et al., 2021; Mita et al., 2009), Southeast Asia had become the initial hotspot for artemisinin resistance. With those drugs, resistant parasites then spread westwards to East Africa, before dispersing widely across the continent and causing a major public health crisis with effects still felt decades later (Trape, 2001; Murray et al., 2012). Now, a major concern is whether ART-R will follow the same trajectory. As Africa carries 94% of the global malaria burden, with over 233 million cases annually, if a decline in artemisinin efficacy caused widespread ACT failure, the consequences could be catastrophic (Dhorda et al., 2024; Duffey et al., 2021; World Health Organization, 2023). Alarmingly, reports of reduced therapeutic efficacy have been increasing in East and Northeast Africa since 2019 (Balikagala et al., 2021; Assefa et al., 2024; Conrad et al., 2023; Uwimana et al., 2020). Although ACTs remain largely effective, several countries have reported efficacies below 90%, including Angola, the DRC, Burkina Faso, Uganda, and Tanzania (Ishengoma et al., 2024; Moriarty et al., 2021; Dimbu et al., 2021; Ebong et al., 2021; Gansané et al., 2021). Monitoring and preventing the spread of ART-R is therefore a major global health priority.

### Monitoring ART-R

ART-R can be monitored using several methods, each of which has their own costs and benefits (Nsanzabana et al., 2018). These can include therapeutic efficacy studies (TES), randomised clinical trials which assess treatment outcomes for ACTs in patients over several weeks and provide crucial evidence for shaping antimalarial policies (Nsanzabana et al., 2018; World Health Organization, 2009). Notably, TES assesses the efficacy of both artemisinin derivatives and their partner drugs, with parasite clearance half-life serving as a specific measure for ART-R (Rosenthal, 2021). Surveillance of ART-R can also involve *in vitro* assays to test for phenotypic resistance, by exposing parasites to varying concentrations of antimalarials to assess their susceptibility (World Health Organization, 2009). Alongside these methods, genomic surveillance has emerged as a key strategy for monitoring ART-R.

Genomic surveillance of ART-R was made possible by the identification of mutations in the *kelch13* gene as the primary determinant of ART-R. Initial evidence for their role was provided by *in vitro* experiments, which associated mutations in the propeller domain of the Kelch13 protein with higher survival rates of ring-stage parasites during drug exposure (Ariey et al., 2014). Gene editing experiments then confirmed this by demonstrating that artemisinin-susceptible parasites could be made resistant by introducing ART-R-associated *kelch13* mutations (Straimer et al., 2015). Further studies, including genome-wide association analysis and selection scans, later identified additional *kelch13* mutations which were strongly associated with resistance (Cerqueira et al., 2017; Anderson et al., 2017). Today, the WHO maintains a list of 23 *kelch13* mutations linked to ART-R, all within the BTB/POZ or propeller domain, which are classified as either validated or candidate mutation depending on the strength of their association with ART-R (Supplementary file 1; World Health Organization, 2022b). While *kelch13* remains the primary and most well-studied molecular marker, other loci, including *pfcoronin*, *ubp1,* and *pfap2mu*, have also been implicated in artemisinin partial resistance and may contribute through complementary mechanisms (Demas et al., 2018; Ndiaye et al., 2021; Henriques et al., 2014).

Genomic surveillance of *kelch13* markers has since become a scalable method for monitoring ART-R prevalence at population levels and has been increasingly implemented in malaria endemic countries (Ndiaye et al., 2021). Although the method does not directly measure patient outcomes, it can be particularly informative when combined with therapeutic efficacy or *in vitro* work (Yeka et al., 2019; Ippolito et al., 2020). Given the wide applicability of genomic surveillance, several strategies now exist to monitor ART-R molecular markers over time and across regions, including targeted amplicon and whole genome sequencing, as reviewed in Neafsey et al., 2021. These methods have enabled the collection of extensive datasets on prevalence of *kelch13* markers at population levels (Abdel Hamid et al., 2023). For example, Kagoro et al., 2022 and Kayiba et al., 2021 each characterised the spatial and temporal spread of ART-R at continental scales, underscoring the effectiveness of genomic surveillance for measuring ART-R. While these studies provided valuable insights, a global analysis of ART-R spread utilising publicly available data remains elusive. This is because although extensive surveillance data on *kelch13* markers is available, it is distributed across numerous studies and open databases. Synthesising this information on a global scale would significantly enhance our understanding of the worldwide distribution and dynamics of ART-R.

In this review, we offer a global perspective on the emergence, spread, and potential future developments of ART-R. We consolidate publicly available data on *kelch13* mutations into a single resource–encompassing 112,933 samples–to provide the most comprehensive overview to date. Our global summary then traces the history and current status of ART-R, providing detailed analyses of its spread in Southeast Asia and East/Northeast Africa. By integrating both literature and existing data, we then articulate several potential scenarios for the future trajectory of ART-R in Africa. Finally, we examine the critical role of genomic surveillance in advancing effective malaria control strategies.

## Global overview of *kelch13 markers* in 112,933 samples

We compiled a global dataset of publicly available *kelch13* mutation data for 112,933 samples collected between 1980 and 2023, from 73 countries (see Methods). These data and metadata were compiled from three sources: the MalariaGEN Pf7 release (Abdel Hamid et al., 2023), the Worldwide Antimalarial Resistance Network (WWARN) Artemisinin Resistance Molecular Surveyor, and a literature search. Mutations were annotated based on their artemisinin resistance status using the most recent WHO guidelines: either ‘validated’, ‘candidate’, or ‘no status’ (Supplementary file 1). Parasites with the 3D7 reference *kelch13* sequence or the A578S mutation were classified as artemisinin-susceptible (World Health Organization, 2022b). Throughout this work, we refer to all BTB/POZ and propeller mutations (codons 349–726) as ‘propeller mutations’. Samples from each country were assigned to one of thirteen broader populations, allowing us to assess trends at continental scales (see Methods). These populations included: South America, West Africa, Central Africa, North Africa, Northeast Africa, East Africa, Southern Africa, Western Asia, Eastern South Asia, Far-Eastern South Asia, Western Southeast Asia, Eastern Southeast Asia, and Oceania.

### Global geographic overview

**Figure 1.**
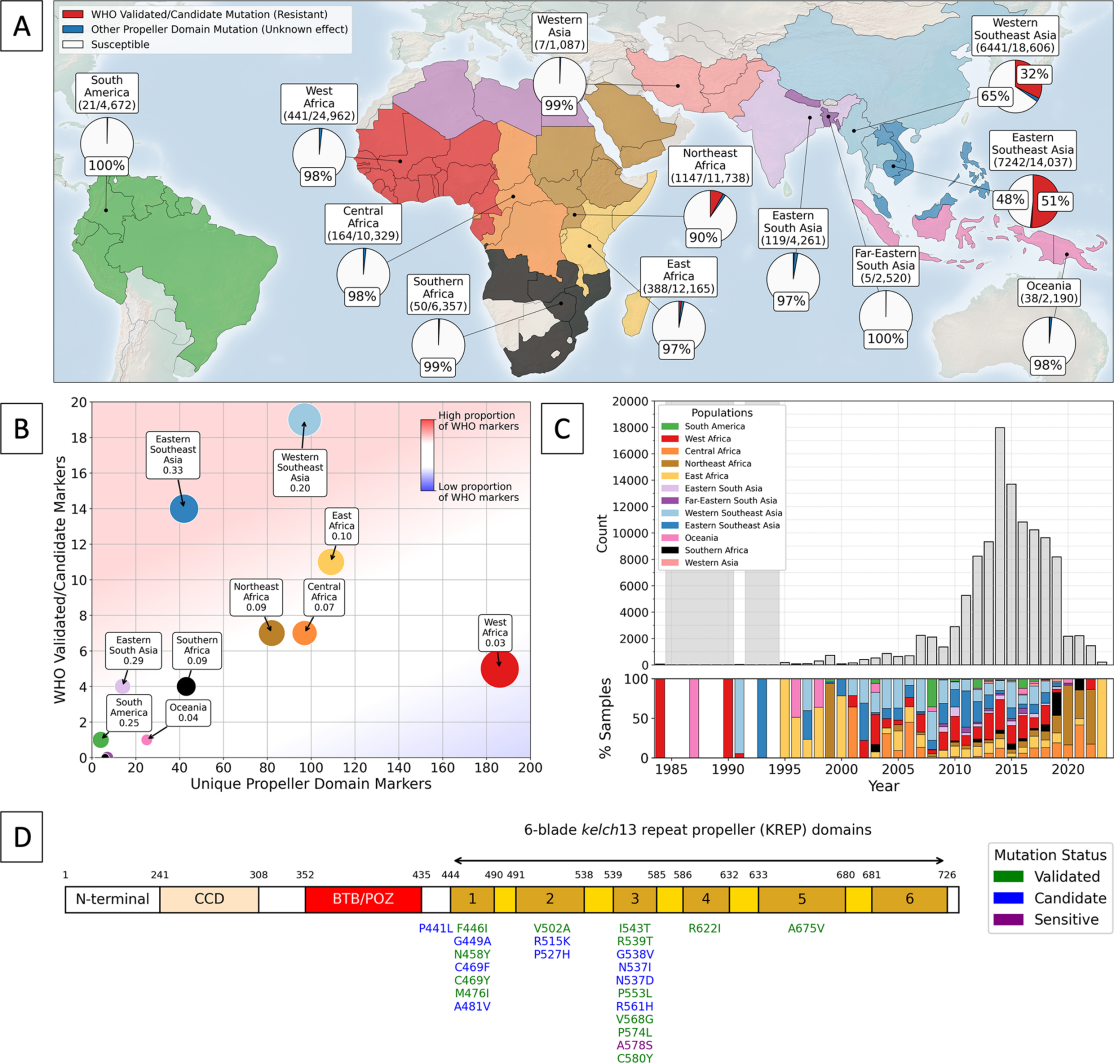
Global trends in *kelch13* propeller mutations over time. Panel A shows the distribution of *kelch13* mutations globally for samples collected between 1980 and 2023. Countries from which samples were included are coloured according to their geographic population assignment. All populations are labelled with a fraction, denoting the number of samples with any *kelch13* propeller mutation (excluding A578S, which is known to be artemisinin susceptible) over the total number of samples from each population. No WHO mutations were detected in the North African samples. For each population, pie charts show the proportion of samples with a WHO validated/candidate mutation in red, the proportion of any other propeller domain mutation in blue, and the proportion of samples with a 3D7 reference *kelch13* sequence or A578S mutation in white. Proportions of each category at 25% or more are labelled. Panel B shows the number of unique *kelch13* propeller domain markers for each population against their total number of unique WHO validated/candidate mutations. Populations are labelled with their ratio of WHO validated/candidate markers to the total number of unique propeller domain markers. Populations falling in the blue shaded area have fewer WHO validated/candidate markers relative to their total count of *kelch13* mutations than populations falling in red areas. The upper plot of panel C shows the total number of samples over time. Grey areas denote years with fewer than 25 samples. The bottom panel shows the proportion of samples from each population for each year, with years with no samples coloured white. Note the limited number of samples from Southeast Asia after 2019, which coincided with an increased proportion of resistant samples in East and Northeast Africa. Panel D illustrates a schematic representation of the *kelch13* protein (residues 1–726), with functional domains—including N-terminal, coiled-coil, BTB/POZ, and six-blade *kelch13* repeat propeller (KREP) domains—annotated using InterProScan (version 5.73). WHO-validated (green) and candidate markers (blue) are mapped to their respective amino acid positions. Figure 1—source data 1.*kelch13* genotypes and sample metadata for 112,933 *Plasmodium falciparum* isolates.

Across the samples, we observed 492 unique, non-synonymous mutations in the BTB/POZ and propeller domains of *kelch13* (Supp. Dataset S2). Most samples had the 3D7 reference sequence (85.5%, n=96,574), with the most common mutation being the WHO validated marker C580Y (6.5%, n=7,307). The other most common mutations were all WHO validated/candidate markers: F446I (1.5%), R561H (0.7%), P441L (0.7%), and R539T (0.4%). Southeast Asia had the highest proportion of samples with a *kelch13* propeller mutation (Figure 1A). For example, over half of samples from Eastern Southeast Asia (52%) had a *kelch13* propeller mutation, with over 98% of these having a WHO validated/candidate marker. Similarly, in Western Southeast Asia, 35% of samples had a *kelch13* propeller domain mutation, with 94% of these having a WHO validated/candidate mutation (Figure 1A). These populations also showed the highest diversity of WHO validated/candidate markers relative to unique *kelch13* propeller mutations (Figure 1B).

The East and Northeast African populations had an increased frequency of WHO validated/candidate markers (Figure 1A). For example, 10% of Northeast African samples had a *kelch13* propeller mutation, ~80% of which were WHO validated/candidate markers. Similarly, in East Africa, 4% of samples had a *kelch13* propeller mutation, and ~25% of these were WHO validated/candidate markers for ART-R (Supplementary file 3). The East and Northeast regions of Africa also had a high number of WHO validated/candidate mutations relative to unique *kelch13* propeller mutations (Figure 1B, Supplementary file 3). In contrast, the proportion of mutant samples was low in other areas of Africa (Figure 1A). In West and Central Africa, for example, mutant samples made up just 2% of the total samples, and this was even lower in Southern Africa (~1%). Similarly, the proportion of samples with a WHO validated/candidate mutation in West, Central, and Southern Africa was also low, typically below 0.13% (Supplementary file 3). Even though West Africa was the largest study population and had the largest number of unique *kelch13* propeller mutations (n=185), only five unique WHO validated/candidate mutations were observed there (Supplementary file 3). Together with lower overall frequencies of WHO mutations, this may imply that there is weaker selective pressure for WHO validated/candidate mutations in West Africa.

Other global populations had low frequencies of *kelch13* mutations and low mutational diversity (Figure 1A). For example, only 0.45% of South American samples had a *kelch13* propeller mutation, and only three unique mutations were reported in the region (Supplementary file 3). One of these was a WHO validated/candidate mutation (C580Y), which was found in 0.41% of all South American samples (Supplementary file 3). Similarly, in Western Asia (the second smallest population), only 0.64% of samples had mutations, none of which had WHO validated/candidate status (Figure 1A). In Eastern South Asia, 3% of all samples had a *kelch13* propeller mutation. In this region, only 13 unique mutations were observed, four of which were WHO validated/candidate mutations, accounting for 0.54% of samples (Figure 1A). In Far-Eastern South Asia, levels of *kelch13* mutation diversity were also very low. Only 0.52% of samples had a *kelch13* propeller mutation, and no WHO validated/candidate mutations were recorded. In Oceania, ~2% of all samples had a mutation in the *kelch13* propeller domain (Figure 1A). While 24 unique *kelch13* propeller mutations were observed in Oceania, only one had WHO validated/candidate status (Figure 1A). For a complete list of mutation metrics in all populations, see (Supplementary file 3).

### Global temporal overview

**Figure 2.**
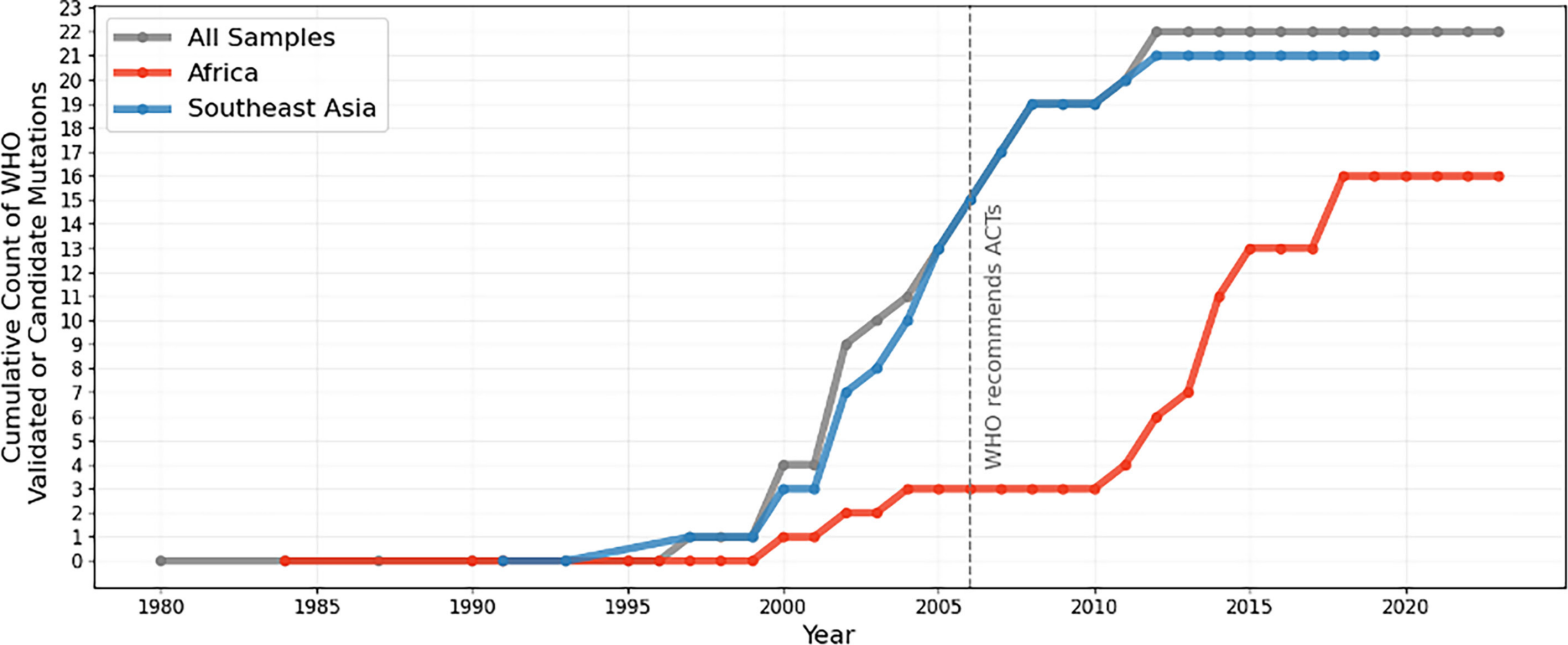
Cumulative count of unique WHO validated/candidate *kelch13* mutations over time across the whole dataset (grey), Southeast Asia (blue) and Africa (red). Figure 2—source data 1.*kelch13* genotypes and sample metadata for 112,933 *Plasmodium falciparum* isolates.

**Figure 3.**
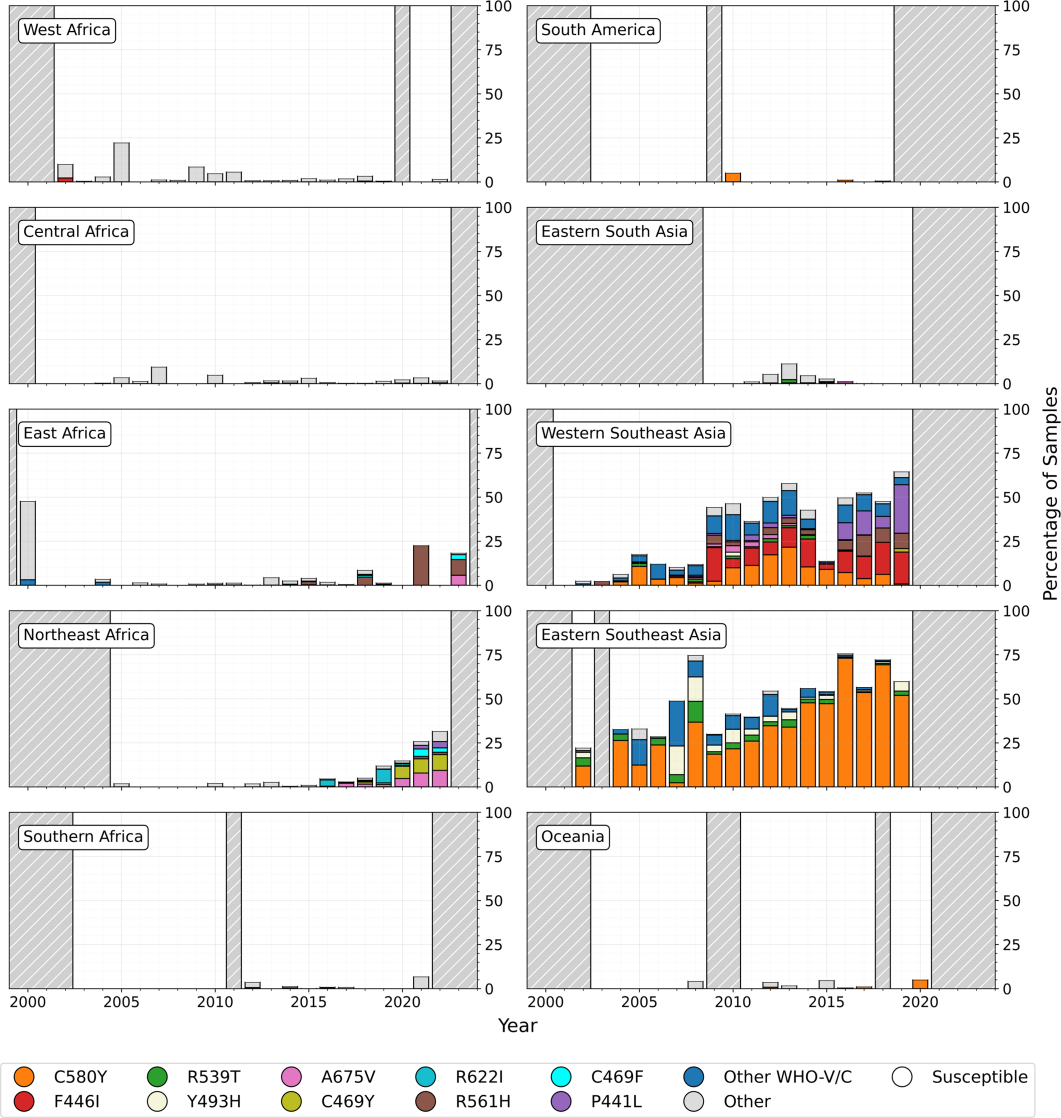
Proportions of *kelch13* mutations over time from 2000 to 2024 for ten populations, with African populations in the left-hand column. Years with fewer than 25 samples are shaded in grey with white stripes. Three populations (North Africa, Western Asia, Far-Eastern South Asia) are not displayed due to lack of data. Several of the most common WHO validated/candidate markers are highlighted. Other WHO validated/candidate markers are denoted by ‘Other WHO-V/C’. Mutations in the *kelch13* BTB/POZ or propeller domain without validated/candidate status are categorised as ‘Other’. The 3D7 reference *kelch13* sequence and the A578S mutation are known to not confer ART-R, so are categorised together as ‘Susceptible’. The high percentage of ‘Other’ mutations in East Africa in 2000 is due to our aggregation of samples from 1996 to 2003 to the median year of 2000 from a single study by de Laurent et al., 2018. Similarly, the percentages of ‘Other’ mutations in West Africa (2005) and Central Africa (2007) result from single studies (Ouattara et al., 2015; Taylor et al., 2015) where multiple different mutations were identified.

**Figure 4.**
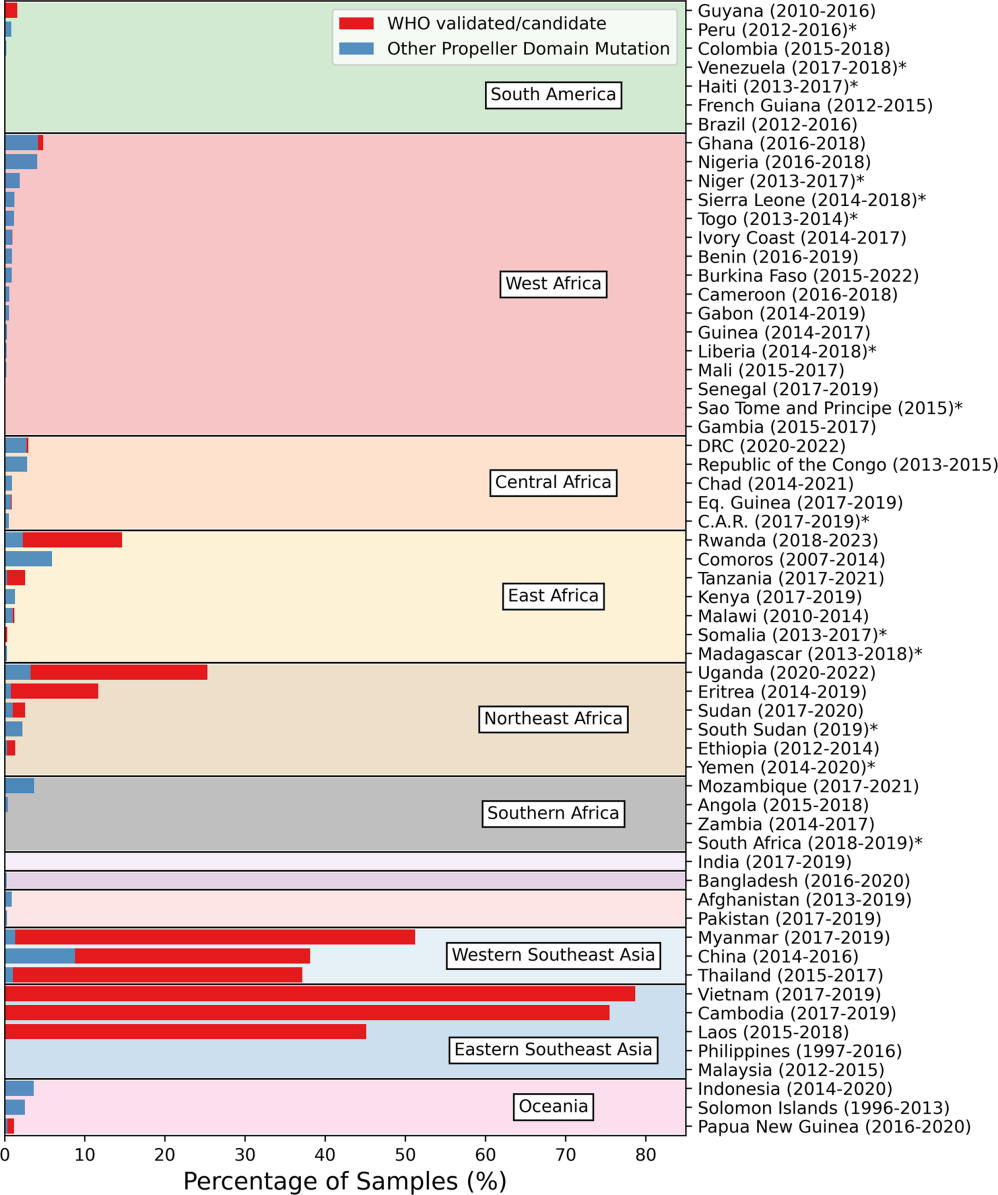
Proportion of samples from each country and geographical population with a WHO validated/candidate *kelch13* mutation (red) or other *kelch13* mutation in the BTB/POZ or propeller domain (blue). Bars show the weighted average percentage of samples from the three most recent observed years with at least 25 samples. Averages were weighted by the total number of samples observed between the years. Date ranges show the range of years for the three most recent years, with at least 25 samples each. Countries with fewer than 3 years with at least 25 samples are marked with an asterisk.

The first documented *kelch13* propeller domain mutation observed in the dataset was from 1991, with the first WHO validated/candidate mutation identified in Thailand in 1997 (R359T). No other ART-R markers were detected before 2000, likely due to limited sampling of *kelch13* propeller mutations pre-2000, with an average of just 138 samples per year (Supplementary file 4). After 2000, sample numbers increased, reaching up to 17,980 in 2014, coinciding with an increase in global ACT use (Figure 1C, Supplementary file 4). The total number of unique *kelch13* mutations and validated/candidate mutations also both rose throughout the 2000s (Figure 2—figure supplement 1). By the time ACTs were introduced as first-line treatments in 2006, over half of the currently known WHO validated/candidate mutations had been observed, predominantly in Southeast Asia (Figure 2). By this time, artemisinin derivatives were already widely used in the region, likely creating an evolutionary pressure for these mutations to emerge and spread (see Section ‘Southeast Asia’). As of the most recent observed years, ART-R-associated mutations have reached high frequency throughout Southeast Asia (Figures 3 and 4).

**Figure 2—figure supplement 1.**
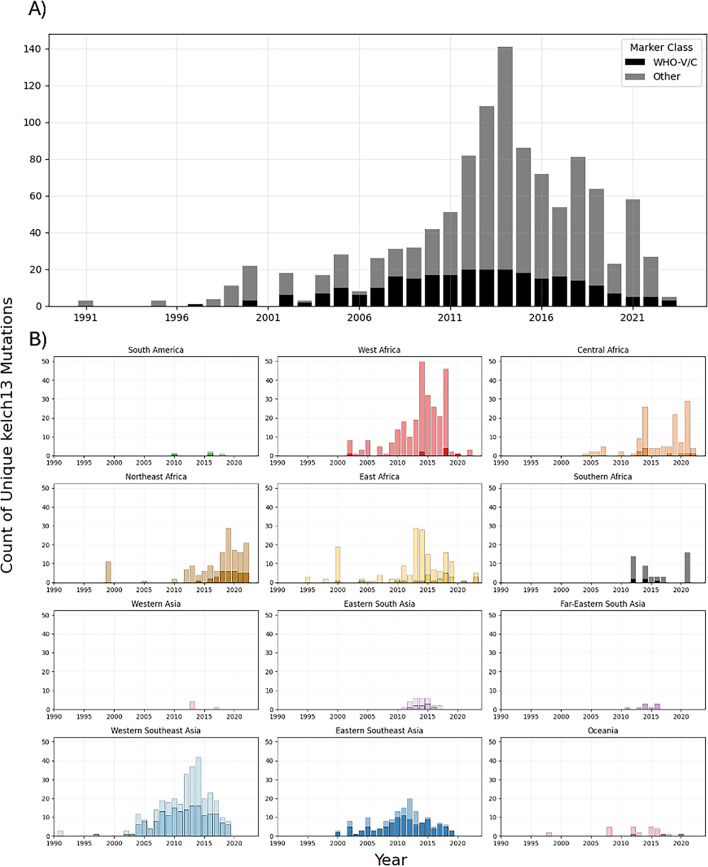
Number of unique WHO validated/candidate *kelch13* mutations and other propeller mutations over time. (**A**) Global overview of unique mutations (**B**) Unique mutations over time split by population. Darker hue = WHO-validated/candidate mutations, lighter hue = other propeller domain mutations. Bars represent stacked mutation counts. North Africa is not shown as no mutations were reported there.

In contrast, ART-R mutations were uncommon in Africa before 2010, with only three unique WHO mutations observed in just two samples each (Figure 2). Several WHO validated/candidate markers then increased in frequency in East and Northeast Africa after 2018 (Figures 3 and 4). For example, seven unique mutations increased in prevalence in Northeast Africa after 2020 (Figure 3), with the WHO-validated mutations A675V and C469Y increasing in frequency between 2020–2022 (from 4.7% to 9.4% and 6.9% to 9%, respectively). Another mutation, C469F, almost tripled in frequency, although overall frequency remained low, increasing from 0.9% in 2020 to 2.5% by 2022. Three WHO validated/candidate mutations were detected in East Africa in 2023: C469F (2.8%), A675V (5.6%), and R561H (declined from 22.5% in 2021 to 8.9% in 2023). In Central Africa, validated/candidate markers P441L, R561H, and C469Y were each detected in single years, although all occurred in fewer than 0.5% of samples. These data support the growing concern that ART-R has begun to emerge in Africa (see Section ‘East and Northeast Africa’).

### Data limitations

While aggregating such a large dataset allowed us to identify trends in the spread of *kelch13* mutations, it also highlighted biases in the available data. For example, sampling has consistently declined in the past decade (Figure 1C). Between 2020 and 2023, the average number of samples was just 1,513–a tenfold decrease compared to 2014 (Figure 1C, Supplementary file 4). Strikingly, there were no data from Southeast Asia in our dataset after 2019, despite the high proportion of mutant samples and variety of *kelch13* mutations in the region before this time. Therefore, trends in ART-R described in this decade are most relevant to Africa. A similar decrease occurred in Southeast Asian samples between 2014 and 2019, although sampling continued. This drop in sample sizes may be explained by a lag in publication of samples after 2019, combined with difficulties in sample collection during the COVID-19 pandemic*,* although this does not explain the decrease before this time. According to the 2023 world malaria report, total funding of malaria control in 2022 was US$3.7 billion, below the estimated total required to meet global technical strategy targets for malaria control and elimination (World Health Organization, 2023). It is possible that this inadequate funding contributed to reduced sampling; however, more evidence would be needed to establish this.

There were also geographical biases in the dataset, with most samples coming from Africa (58%) and Southeast Asia (29%). This geographic bias reflects Africa’s status as a region with a high *P. falciparum* malaria burden and Southeast Asia’s role as the epicentre of ART-R. As a result, these regions have generated a significant proportion of the data relevant to this review. Thus, the trends described here are most applicable to these regions, despite sampling from a global range. This bias was especially pronounced between 2014 and 2016, with most samples coming from West Africa during this time (Figure 1C, Supplementary file 4). There were also biases within continents; for example, West Africa made up 52% of African samples in 2014, but by 2019, this was just 5%. Similarly, in Northeast Africa, these proportions were 9% in 2014 and 25% in 2019. Since 2019, there was no year in which all of the regions most impacted by malaria were adequately represented. At a smaller scale, we also saw an under-representation of some countries within populations (Supplementary file 2).

A further source of potential bias is in the different SNP detection methods used across data sources, with DNA sequencing using capillary electrophoresis (also known as Sanger sequencing) accounting for 77.01% of samples, while next-generation sequencing (NGS) methods represented 22.10% (Source Data S1). A key limitation of dideoxy sequencing is its reduced sensitivity in detecting low-frequency variants, particularly in highly mixed infections. This limitation may have led to an underestimation of some variants, especially in settings with high within-host diversity.

Sampling biases can arise from various sources, including the variation in location or number of health facilities between regions and differing sampling regimes between national control programmes (Mayor et al., 2023). Going forwards, having systematic, longitudinal sampling in Africa that is made publicly available as soon as possible will be crucial for providing robust insights into the effectiveness of ACTs. While aggregating such a large dataset still allows us to make robust comparisons within and between populations, these biases mean the findings need to be interpreted carefully.

## Spread of artemisinin partial resistance

In the previous section, we highlighted how markers of ART-R have been found across the globe, but until recently, had been mostly localised in Southeast Asia. Concerningly, several WHO validated/candidate mutations have now emerged in parts of Africa, with potentially widespread consequences for human health. Here, we provide detailed case studies for Southeast Asia and East/Northeast Africa, focusing on how resistance-associated *kelch13* markers emerged and spread, summarising their current frequencies, and highlighting key questions on their geographical distributions. We then discuss *kelch13* substitutions observed in other regions, including West and Central Africa, South America, and Oceania.

### Southeast Asia

#### How did *kelch13* mutants initially emerge and spread in Southeast Asia?

**Figure 5.**
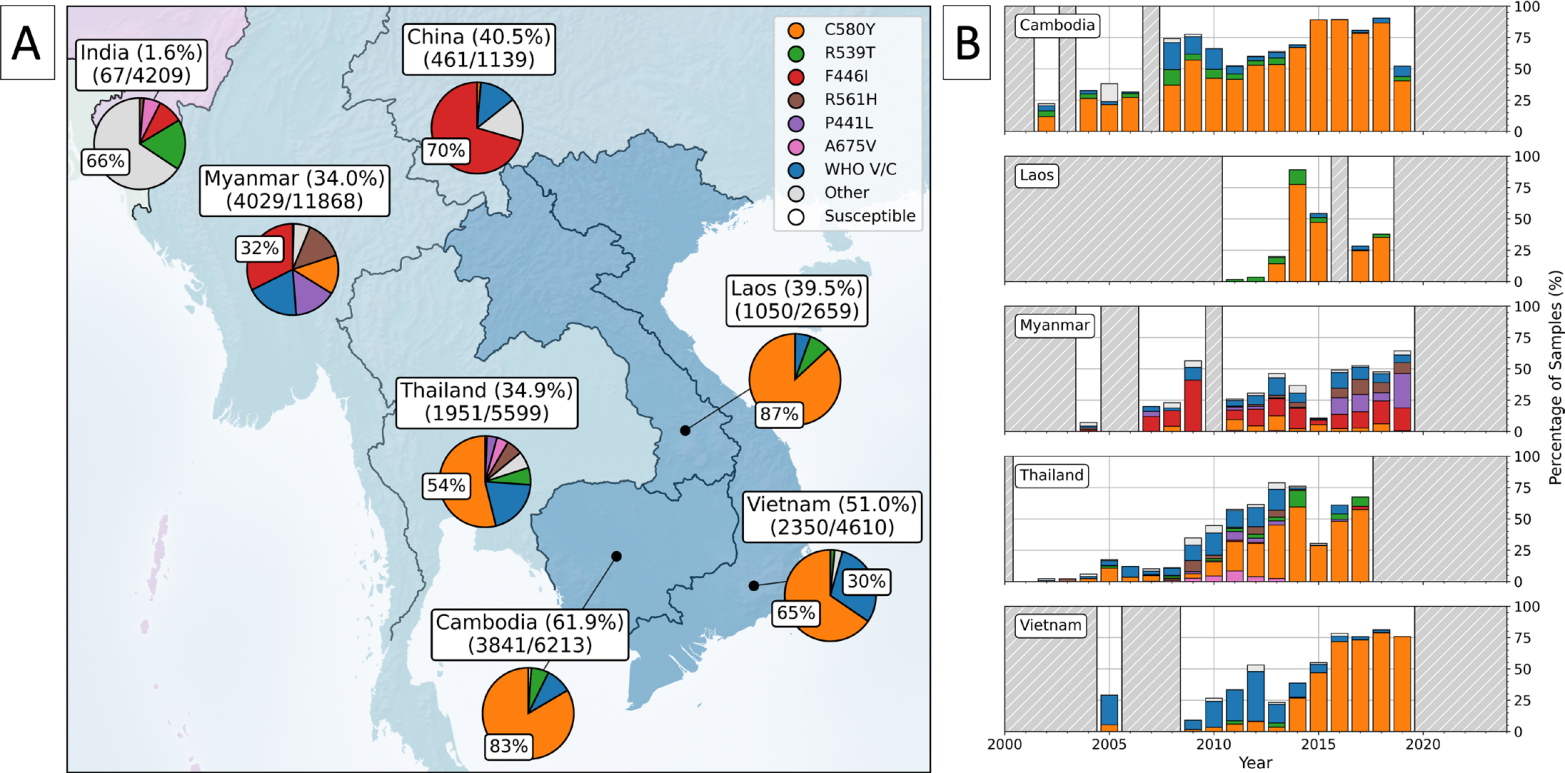
Regional prevalence and temporal changes of *kelch13* propeller mutations in Southeast Asia. Panel (**A**) shows the distribution of *kelch13* propeller mutations across Southeast Asia. The percentage of samples with any *kelch13* propeller mutation are included in the country labels, above the number of samples with any *kelch13* propeller mutation and the total number of samples collected for each country, respectively. Among only the samples with any observed *kelch13* propeller mutation, pie charts show the proportions with markers of interest, where proportions above 25% are labelled accordingly. ‘Other’ denotes low-frequency mutations in the propeller domain which are not WHO validated/candidate markers, aggregated into a single category. Samples with the 3D7 reference sequence for *kelch13* or A578S are denoted as ‘Susceptible’ (World Health Organization, 2022b). Panel (**B**) shows all samples collected over time for each country as a stacked bar chart, where the proportion of samples with each marker is coloured. Years with fewer than 25 samples are highlighted with grey dashed lines.

Artemisinin combination therapies have been used extensively across Southeast Asia since the mid-1990s, where they were quickly adopted by national governments seeking alternatives to drugs such as chloroquine, which had developed widespread resistance. Thailand, for example, adopted ACTs in 1995, shortly followed by Cambodia in 2000, Myanmar in 2002 and Vietnam in 2003 (Hassett and Roepe, 2019; Satimai et al., 2012). While ART-R was only discovered in Southeast Asia in 2008, the proportion of samples with a validated/candidate mutation had in fact already been high by 2006 (Figure 5; Kagoro et al., 2022). ART-R associated *kelch13* mutations then emerged independently across several locations in Southeast Asia, before a hard selective sweep drove a multidrug resistant co-lineage to dominance across the region (KEL1/PLA1, containing the C580Y *kelch13* mutation) (Takala-Harrison et al., 2015; Imwong et al., 2017; Imwong et al., 2020; Amato et al., 2018; Hamilton et al., 2019). Analysis of genomic regions flanking the *kelch13* gene suggested this migration occurred several times, with the C580Y mutation initially spreading eastward from Cambodia to Vietnam, and later, from Cambodia to Thailand and Laos around 2013–15 (Takala-Harrison et al., 2015; Imwong et al., 2017). Over the next 10–12 years, the C580Y mutation reached near-fixation frequencies in several of these countries (Figure 5). In contrast, in Myanmar, C580Y emerged and spread independently on a different parasite genetic background (Imwong et al., 2017). This pattern of identical *kelch13* mutations arising independently, alongside migration events between countries, would become a repeated occurrence across Southeast Asia, with several of the most common *kelch13* mutations spreading via both mechanisms (Takala-Harrison et al., 2015; Imwong et al., 2017; Imwong et al., 2020; Hamilton et al., 2019).

**Figure 5—figure supplement 1.**
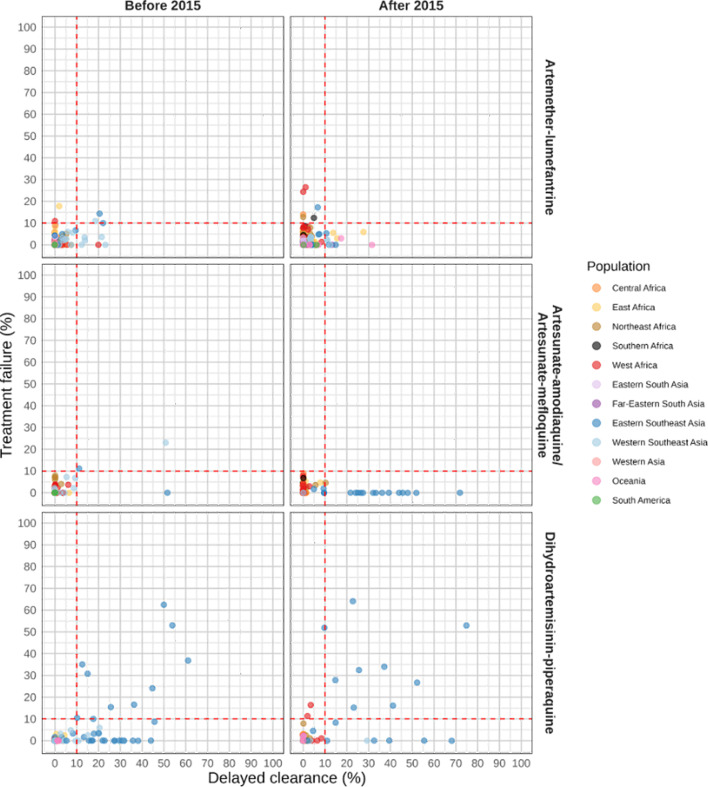
Scatter plot showing the results of TES monitoring three commonly used ACTs across populations, using the data from the WHO Malaria Threats Map (https://apps.who.int/malaria/maps/threats/). The x-axis represents delayed clearance, while the y-axis represents treatment failure rates. Scattered data points are colour-coded by region. Red dashed lines indicate the 10% threshold established by the WHO for policy change.

Genomic epidemiology played a critical role in identifying these patterns of emergence and migration in Southeast Asia. While it was initially thought there had been a single emergence of ART-R in the region, comparison of parasite genetic backgrounds revealed it had in fact emerged independently across several countries (Neafsey et al., 2021; Amato et al., 2018; Verity et al., 2020). This enabled policy makers to focus on slowing local resistance through improved surveillance and access to malaria prevention, rather than focusing solely on preventing parasite migration across borders (World Health Organization, 2015b; Manzoni et al., 2024). Data from these surveillance efforts then became a cornerstone for developing mitigation strategies in the region. One such strategy, the ‘WHO Strategy for Malaria Elimination in the Greater Mekong Subregion’ (2015–2030), leveraged this evidence to achieve significant reductions in overall malaria burden of 77% (World Health Organization, 2015b; Manzoni et al., 2024). This underscores the importance of accurate and consistent molecular surveillance data in formulating effective public health policies and interventions.

#### What is the current distribution and frequency of *kelch13* mutations across Southeast Asia?

The proportion of samples with a WHO validated or candidate *kelch13* substitution remained high as of 2019. Based on the latest data, between 37.9–75.8% of samples had a validated/candidate mutation in most Southeast Asian countries (Figure 5). For example, the proportion of samples with a WHO validated/candidate mutation in Vietnam (2019) and Thailand (2017) was 75.8% and 67.5%, respectively (Supplementary file 5). Similarly, in Cambodia, these frequencies fluctuated between 80% and –90% from 2016 to 2018. In 2019, 52.1% of Cambodian samples had a WHO validated/candidate mutation; however, data for this year were limited to three sites from a single study (Peto et al., 2022). Laos showed a slightly lower frequency of 37.9% (2018), although it exhibited significant year-to-year variability, with a peak of 89% in 2014 (based on 111 samples). In contrast, Myanmar showed both a lower frequency and slower increase in the proportion of samples with a *kelch13* mutation between 2004 and 2019 (Supplementary file 5), although frequencies were still high as of the most recent year (51.5%). Between 1993 and 2019, there were 117 unique *kelch13* mutations present across Southeast Asia, although only 22 of these were observed in more than 25 samples (Table 1). While the C580Y mutation, a WHO-validated marker, was initially only one of several markers to emerge, it later rose to high frequency in many countries, such as Thailand, Laos, Cambodia, and Vietnam.

**Table 1.** Most common *kelch13* mutations across Southeast Asia, where mutations occur in at least 25 samples across the two populations (western and eastern Southeast Asia). Countries represented in this summary are Myanmar, China, Thailand, Cambodia, Laos, and Vietnam. Total refers to the total number of samples from these countries with each marker.

Marker	WHO status	Total	Proportion (%)
3D7 reference sequence	-	18,958	58.08
C580Y	validated	7,271	22.27
F446I	validated	1,644	5.04
P441L	candidate	672	2.06
R561H	validated	666	2.04
R539T	validated	478	1.46
Y493H	validated	437	1.34
I543T	validated	391	1.20
G449A	candidate	355	1.09
P574L	validated	292	0.89
P553L	validated	226	0.69
M476I	validated	173	0.53
N458Y	validated	140	0.43
G538V	candidate	126	0.39
A675V	validated	95	0.29
G533S	no status	86	0.26
N537I	candidate	49	0.15
A676D	no status	44	0.13
V568G	candidate	38	0.12
M562I	no status	35	0.11
T474I	no status	31	0.09
C469F	candidate	30	0.09
E461G	no status	27	0.08

As of 2019, C580Y remains dominant, with the majority of samples with any *kelch13* mutation showing this marker. For instance, 83% of all samples from Cambodia with a *kelch13* mutation had C580Y. A similar trend was observed in Thailand (54%) and Vietnam (65%). While other *kelch13* substitutions were circulating in these countries, such as R539T and R561H, these were at much lower frequencies. Notably, Thailand showed a larger diversity of other markers than Cambodia, Vietnam, or Laos. This is most likely due to the malaria-free corridor which separates parasite populations in east and west Thailand. Eastern Thailand tends to have higher proportions of C580Y, while western Thailand more closely reflects the diversity in bordering Myanmar (Kobasa et al., 2018). In Myanmar, several unique *kelch13* mutations were circulating at moderate frequencies in 2019, with no single substitution reaching fixation. C580Y is not the dominant mutation in Myanmar, where mutations such as F446I, R561H, and P441L have all continued circulating (Bonnington et al., 2017). This pattern of several circulating markers has remained consistent between 2004 and 2019, whereas countries such as Thailand and Laos saw C580Y overtake others during the same time period. In total, of the 22 most common *kelch13* mutations, 8 of these were unique to Myanmar and China within Southeast Asia, including: E461G, A675V, M562I, F446I, A676D, G538V, R561H, P441L.

The high frequency of *kelch13* mutations in Southeast Asia has translated into increased negative clinical outcomes, evidenced by delayed clearance rates above 10% in TES (Supplementary files 6). We obtained the results of studies which monitor the therapeutic efficacy of common ACTs from the WHO Malaria Threats Map (Manzoni et al., 2024, Figure 5—figure supplement 1). Out of 94 studies across Southeast Asia, 52 (55%) reported delayed clearance rates (>10%) for dihydroartemisinin-piperaquine (DHA-PPQ) treatment, with 18 (19%) reporting treatment failure rates over 10%. Artesunate-mefloquine (AS-MQ) paints a similar picture: of 38 studies, 21 (55%) reported delayed clearance rates, with 2 (5%) reporting treatment failure rates over 10%. In contrast, artemether-lumefantrine (AL) has shown slightly better outcomes, with only 15 of 131 studies (11%) reporting delayed clearance rates of >10%, and four (3%) reporting treatment failure rates >10%. This lower delayed clearance rate for AL may be attributed to the fact that AL has only been a first-line treatment in Laos and Myanmar, where ART-R is lower (World Health Organization, 2023; World Health Organization, 2015b; World Health Organization, 2021; Jacob et al., 2021). The high frequency of delayed clearance and treatment failure indicates the growing challenge in malaria treatment using ACTs in Southeast Asia.

These findings underscore the need for ongoing genomic surveillance to establish the composition and frequencies of circulating parasite populations in Southeast Asia. However, data from the region are limited by the lack of systematic, longitudinal sampling, without which it is difficult to properly estimate the frequencies of resistance (Mayor et al., 2023). For example, in our data, no information was available for Southeast Asian countries after 2019. Additionally, many countries showed considerable between-year variation in the proportion of samples with *kelch13* mutations, which may be due to a disproportionately large number of samples from a single study or region and inconsistent sampling. One major challenge for surveillance is that it becomes more challenging to collect samples as overall numbers are driven down, as is the case in Southeast Asia. While systematic genomic surveillance is still key to interpreting the frequencies of *kelch13* mutations, going forwards, additional strategies may also be required to account for lower case numbers. These could include enhanced collaborations and data sharing to pool data, and compensatory statistical techniques like time-aggregated sampling.

#### Did fitness costs affect the spread of kelch13 mutations across Southeast Asia?

Experimental studies have assessed the fitness costs of *kelch13* mutations using various methods, including growth and competition assays, as well as measurements of metabolic and transcriptional responses (see Box 1 for an overview). Although validated and candidate mutations have so far been described in this review as conferring a unified ‘resistant’ phenotype, in reality, individual mutations can differ in their effects on both parasite fitness and clearance rate, and these effects may vary further depending on the context in which the parasite is treated with artemisinin (Behrens et al., 2023; WWARN K13 Genotype-Phenotype Study Group, 2019).

Box 1. **Fitness costs associated with ART-R.**

Fitness costs could significantly impact the spread of ART-R. In the presence of the drug, resistant parasites have a selective advantage, making them more likely to transmit between individuals and spread within a population (White, 2004). However, if there are fitness costs to being resistant, then in the absence of the drug, resistant parasites would be outcompeted by susceptible genotypes, making them more likely to remain at low frequency (Rosenthal, 2013).

Numerous *in vitro* studies have demonstrated fitness costs associated with *kelch13* mutations, such as decreased parasite blood stage growth rate (Straimer et al., 2017) and competitive growth disadvantage (Stokes et al., 2021; Nair et al., 2018; Mathieu et al., 2020). Delayed parasite clearance under artemisinin exposure is linked to decreased haemoglobin endocytosis, limiting the parasite’s ability to utilise important amino acids (Bunditvorapoom et al., 2018). *kelch13* mutant parasites also have lower heme levels during the trophozoite stage, which may cause growth defects (Heller and Roepe, 2018b; Heller et al., 2018a). *kelch13* mutations may also affect stability of the protein, leading to transcriptional stress responses and cell development issues (Mok et al., 2015; Behrens et al., 2024; Yang et al., 2019). Moreover, different *kelch13* mutations can confer differing levels of ART-R and fitness costs. For example, C580Y and R561H show similar increased survival under drug treatment *in vitro*, but differing fitness costs (Nair et al., 2018). The F446I mutation, common in Myanmar, provides a small survival advantage during drug treatment, but lower competitive growth *in vitro* than C580Y (Stokes et al., 2021). While other regions of the genome have also been associated with decreased clearance rate for artemisinin (Cerqueira et al., 2017; Mukherjee et al., 2017), these mechanisms are complex and require further experimental validation of potential fitness costs (for a review, see Pandit et al., 2023).

The presence of these costs is unsurprising given the highly conserved nature of the kelch13 propeller domains, yet may have important consequences for selection for ART-R (Ariey et al., 2014; Miotto et al., 2015). Fitness costs may be one reason why *kelch13* mutations are typically observed in isolation, rather than multiple mutations within a single parasite. This effect was seen in our aggregated dataset, where only one double *kelch13* propeller mutation (M579T/N657H) occurred in a single study (Mishra et al., 2017). However, as neither M579T nor N657H was found individually or together in other studies, this double mutation may have arisen from laboratory cross-contamination or a mixed-strain infection.

Interestingly, differences in the clearance rate and fitness effects of different *kelch13* mutations are correlated with their geographic distribution across Southeast Asia (Straimer et al., 2015; Stokes et al., 2021). For example, although the R539T mutation has a larger effect in ring-stage survival assays (RSA) than C580Y, it also generates a larger fitness cost *in vitro* (Ashley et al., 2014; Straimer et al., 2015; Stokes et al., 2021). This difference corresponds closely with their frequency in Southeast Asia, with C580Y generally being the more prevalent substitution across much of Eastern Thailand, Laos, and Cambodia (Figure 5). Another example is the F446I mutation, which confers a lower degree of resistance than C580Y, but also a lower fitness cost in competition assays (Ashley et al., 2014; Stokes et al., 2021). This again correlates with their relative geographical distributions, with C580Y more common across Eastern Southeast Asia, while F446I is more common in parts of Myanmar and Western Thailand (Figure 5). Interestingly, there is known to be increased transmission, lower drug pressure, and higher competition between parasites in Myanmar relative to other areas of Southeast Asia, suggesting the lower fitness cost of the F446I mutation may provide a selective advantage over C580Y in these areas (Imwong et al., 2017; Imwong et al., 2020). This may have resulted in several mutations with lower fitness costs being more strongly selected for than mutations which cause greater ART-R, potentially explaining the relatively stable frequencies of several mutations observed there between 2004 and 2019. In a further example, R561H shows similar survival rates to C580Y under treatment, but a decreased fitness cost *in vitro* (Nair et al., 2018). This could mean R561H has the potential to spread more widely, which is concerning as it has increased in frequency in Myanmar since 2016 (Hamilton et al., 2019; Imwong et al., 2017).

Notably, individual *kelch13* substitutions have been shown to not only have different phenotypic and fitness effects, but these effects can also differ depending on the genetic background of the parasite in question (Stokes et al., 2021; Siddiqui et al., 2021; Stokes et al., 2022). For example, gene-editing studies have demonstrated that after introducing *kelch13* mutations into parasites of Southeast Asian origin, they exert a lower fitness cost and a greater effect on RSA survival than parasites from Africa (Stokes et al., 2021; Stokes et al., 2022). There is also evidence of ‘genetic backgrounds’ which are specifically associated with *kelch13* mutations, and tend to be more common in parasites from areas in Southeast Asia where ART-R has emerged and spread extensively (Cerqueira et al., 2017; Miotto et al., 2015; Uwimana et al., 2021). For example, genomic changes have been identified which may compensate for the fitness costs of the *kelch13* mutations, such as altering nutrient permeable channels in the parasite vacuole membrane and allowing uptake of important amino acids. These backgrounds are more common in Southeast Asia, which could be one possible reason why ART-R emerged and spread in this region so quickly, alongside early and widespread ACT use (Mesén-Ramírez et al., 2021). Although speculative, these effects could mean resistance mutations are more likely to appear (and therefore spread) on certain genetic backgrounds, or in combination with important genetic changes (Cerqueira et al., 2017; Stokes et al., 2021; Miotto et al., 2015). While other regions of the genome have also been associated with decreased clearance rate for artemisinin, information on how these mechanisms may interact with fitness costs is more limited (for a review, see Miotto et al., 2024).

As novel *kelch13* mutations emerge and spread, understanding their fitness costs across different geographical regions and in relation to parasite genetic backgrounds may be crucial. This understanding will require assessing fitness costs through *in vitro* assays and integrating these findings with genomic surveillance to reveal broader patterns. Genomic surveillance may aid in generating models which can predict the spread of resistance given the frequency of certain genetic backgrounds. These patterns also highlight the advantages of whole genome sequencing over targeted amplicon sequencing, as this additional information can be used to inform models using the genetic background of resistant parasites.

### East and Northeast Africa

#### How did *kelch13* mutants initially emerge and spread in East and Northeast Africa?

ACTs have been used extensively across Africa, particularly after 2006 (Newton et al., 2006Supplementary files 6 and 7). By 2008, almost all African countries had implemented ACTs as the primary treatment for malaria, meaning the emergence and spread of ART-R in Southeast Asia around 2008 occurred in tandem with a growing reliance on ACTs in Africa (World Health Organization, 2009). This led to fears that similar mutations could not only arise in Africa, but would result in a wave of resistant parasites. In the last 5 years, those fears are beginning to be realised, with reports of WHO validated/candidate *kelch13* mutations in several countries across East and Northeast Africa. This started with the identification of the R561H mutation in Rwanda in 2019. This mutation was shown to have been circulating in the region since 2013–2015, before increasing to ~13% in 2019, with demonstrated effects on parasite clearance rate (Uwimana et al., 2020; Uwimana et al., 2021). This was then mirrored by the dramatic increase in both A675V and C469Y mutations in Uganda between 2 015 and 2019, which were also associated with delayed parasite clearance (Balikagala et al., 2021). Given the extent of ACT use in Africa over the past two decades, it is unclear why ART-R has only emerged now, around 10–15 years behind Southeast Asia, although it is most likely due to the slower initial rollout of ACTs in Africa, combined with differences in case-by-case drug exposure (Hassett and Roepe, 2019). Other factors, such as higher immunity, intra-host competition, or differences in effective population sizes in Africa may have also slowed ART-R (see Section ‘Scenario 3: Lower frequency and slower spread of ART-R in Africa than Southeast Asia’).

After detecting ART-R associated *kelch13* mutations in Africa, an initial concern was that these mutations had been introduced via gene flow from Southeast Asian parasites, as was the case with resistance to chloroquine and sulfadoxine–pyrimethamine (Wellems and Plowe, 2001; Gregson and Plowe, 2005). However, evidence from genomic surveillance suggests that the *kelch13* mutations observed so far in Africa have emerged independently. For example, ART-R parasites from Uganda are genetically more similar to African parasites than those from Southeast Asia, suggesting an independent African emergence (Ikeda et al., 2018). Similarly, analysis of *kelch13* flanking microsatellites and genome-wide single-nucleotide polymorphisms suggests Ugandan parasites with C469F, C469Y, and A675V form a clade distinct from Southeast Asian parasites, implying a single evolutionary origin and clonal expansion within Africa (Conrad et al., 2023). A further example is A675V, C469Y, and R561H mutants from Uganda, Rwanda, and Tanzania, which have different *kelch13* flanking haplotypes to parasites with these mutations from Southeast Asia, again pointing to a single evolutionary origin which arose independently in Africa (Balikagala et al., 2021; Uwimana et al., 2020; Ishengoma et al., 2024). Together, these analyses show clear evidence of independent emergence of *kelch13* markers in Africa, suggesting local conditions are prompting the emergence and selection of ART-R. Nevertheless, ongoing genomic surveillance will be needed to identify migratory pathways between continents should they occur.

#### What is the current distribution and frequency of *kelch13* mutations in East and Northeast Africa?

**Figure 6.**
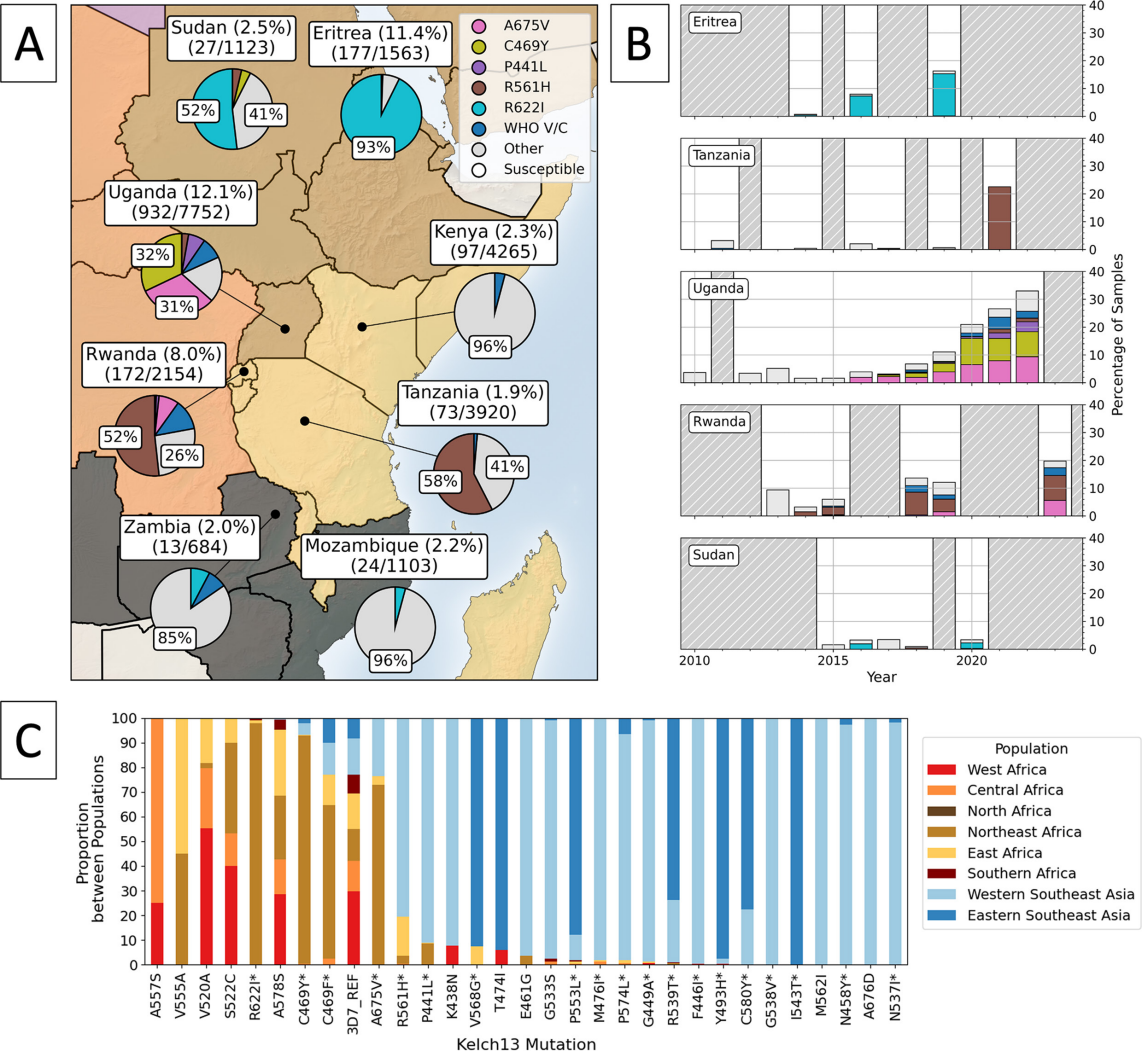
Regional prevalence and temporal changes of *kelch13* propeller mutations in East and Northeast Africa. Panel (**A**) shows the distribution of *kelch13* propeller mutations across East and Northeast Africa. The percentage of samples with any *kelch13* propeller mutation is included in the country labels, above the number of samples with any *kelch13* propeller mutation and the total number of samples collected for each country, respectively. Among only the samples with any observed *kelch13* propeller mutation, pie charts show the proportions with markers of interest, where proportions above 25% are labelled accordingly. ‘Other’ denotes low-frequency mutations in the propeller domain which are not WHO validated/candidate markers, pooled into a single category. Samples with the 3D7 reference sequence for *kelch13* or A578S are denoted as ‘Susceptible’ (World Health Organization, 2022b). Panel (**B**) shows all samples collected over time for each country as a stacked bar chart, where the proportion of samples with each marker is coloured. Years with fewer than 25 samples are highlighted with grey dashed lines. Notably, Uganda and Rwanda have shown a consistent increase in diversity among *kelch13* mutations between 2016–2023. (**C**) shows all mutations with at least 25 samples across Africa and Southeast Asia on the x axis. Coloured bars show which subpopulation these samples came from across Africa and Southeast Asia. WHO validated or candidate mutations are suffixed with an asterisk.

As of 2024, WHO validated/candidate mutations have been identified in several countries across East and Northeast Africa, including Uganda, Rwanda, Sudan, Eritrea, Tanzania, Kenya, and Ethiopia (Fola et al., 2023; Figure 6). Concerningly, the frequency of these mutations has increased dramatically since 2018, particularly in Uganda, Rwanda, Tanzania, and Eritrea. In 2022, 26% of samples from Uganda had a WHO validated/candidate mutation, an increase from ~1% in 2013. Similarly, 17% and 15% of samples from Rwanda and Eritrea had a WHO mutation in 2023 and 2019, respectively. Tanzania also had a high proportion of parasites with WHO mutations in 2021 (23%), increasing from ~0.1% to 0.2% between 2017 and 2019. However, Tanzanian samples from 2021 were all from close to the borders with Uganda and Rwanda, which have high levels of ART-R, suggesting a possible geographic sampling bias. Sudan showed a small proportion of parasites with a WHO mutation, reaching 2.2% in 2020. Similarly, Kenya, DRC, Zambia, and Mozambique each showed a small proportion of samples with a WHO mutation, although the total frequencies of samples with any propeller domain mutation in these countries remained ~4% or less. Concerningly, these trends are strikingly similar to those observed in Southeast Asia around 2008, where the initial emergence of validated/candidate mutations was followed by exponential increases in the frequency of resistant parasites over a decade.

Alongside an increase in WHO validated/candidate mutations, many East and Northeast African countries also had an increase in frequency of propeller domain *kelch13* mutations which are not assigned WHO validated/candidate status. These countries included Kenya, Rwanda, Tanzania, Eritrea, South Sudan, Sudan, and Uganda. The phenotypic effects of these mutations are unclear, but in some cases, *in vitro* evidence suggests they could have phenotypic effects on artemisinin clearance rates (WWARN K13 Genotype-Phenotype Study Group, 2019; Mukherjee et al., 2017). Tracking the changes in frequency of these mutations is important, as they may in fact provide some degree of ART-R.

As of 2024, we have not seen any single WHO validated/candidate mutation rise to prominence in East or Northeast Africa, although this may occur in coming years - as with C580Y in Southeast Asia. In the past 5 years, several WHO validated/candidate markers have circulated simultaneously, with individual mutations mostly localised to specific countries within the region, similar to patterns seen in the early period of ART-R in Southeast Asia (Figure 6 and Table 2). For example, the most common WHO validated/candidate mutation, A675V, has risen to high frequencies in Uganda and Rwanda (9.4% in 2022 and 5.6% in 2023, respectively), but has not been observed in neighbouring countries. Similarly, Rwanda, and Tanzania have a high frequency of parasites with the R561H mutation (8.9% in 2023 and 22.5% in 2021), but this mutation is uncommon elsewhere. A third example is R622I, which was the most common mutation in Eritrea (15.2% in 2019) and Sudan (2% in 2020), but rare outside Northeast Africa. Several of these mutations have increased in frequency over the past 5 years, with A675V, C459Y and P441L all increasing in frequency in Uganda since 2018. A similar situation occurred in Rwanda, where three validated/candidate mutations increased in frequency between 2019 and 2023; A675V from 1.5% to 5.6%, C469F from 1.5% to 2.8%, and R561H from 4.5% to 8.9%. The emergence and spread of different *kelch13* mutations in separate regions of East and Northeast Africa suggests they are emerging independently in response to local conditions, rather than migrating between areas (Assefa et al., 2024; Abera et al., 2021).

**Table 2.** Most common *kelch13* mutations across East and Northeast Africa, where mutations occur in at least 25 samples. Countries represented in these populations are Burundi, Comoros, Kenya, Madagascar, Malawi, Rwanda, Somalia, Tanzania, Eritrea, Ethiopia, Saudi Arabia, South Sudan, Sudan, Uganda, and Yemen. Note A578S is not associated with ART-R (World Health Organization, 2022b). Total refers to the total number of samples from these countries with each marker.

Marker	WHO status	Total	Proportion (%)
3D7 reference sequence	-	22,219	92.95
A675V	validated	306	1.28
C469Y	validated	301	1.26
R622I	validated	184	0.77
R561H	validated	158	0.66
A578S	not associated	149	0.62
C469F	candidate	97	0.41
P441L	candidate	65	0.27
V555A	no status	29	0.12

Interestingly, the WHO validated/candidate *kelch13* mutations which have arisen in East and Northeast Africa are different to those found in Southeast Asia (Figure 6C). For example, the most common *kelch13* mutations in Southeast Asia are C580Y and F446I, but these are uncommon in Africa, with C580Y only observed in seven samples across the entire continent. Other common substitutions in Southeast Asia were also rare in Africa, including R539T, P574L, F446I, and G449A, all with fewer than five samples. Similarly, of the most common WHO validated/candidate markers in Africa (A675V, C469Y, R622I, R561H, and C469F), only R561H was found regularly in Southeast Asia. This could be due to differences in ACT use between continents. For example, artemether-lumefantrine is the most common treatment for malaria in Africa, but in Southeast Asia, dihydroartemisinin-piperaquine and artesunate-mefloquine are used more often (Tun et al., 2018; World Health Organization, 2022a; Supplementary file 7). Theoretically, these differences could promote different *kelch13* mutations, because their partner drugs are associated with selection for different genetic backgrounds with differing resistance mutations. For example, R539T has been associated with mefloquine-resistant parasites, whereas C580Y has been associated with piperaquine-resistant parasites, because changes in *mdr1* and *plasmepsin2/3,* respectively, alter the fitness costs of *kelch13* mutations in the presence of these different drugs (Parobek et al., 2017). It is also possible that differences in the phenotypic effects of the substitutions, alongside variation in transmission rates, fitness costs or genetic backgrounds in Africa, have selected for different *kelch13* mutations relative to Southeast Asia, although this remains speculative.

#### Why is ART-R centred in Eastern and Northeastern Africa?

It is unclear why WHO validated/candidate mutations have arisen in Northeast and East Africa first, or why we have not seen a wider spread of these mutations across Africa, despite extensive ACT use. One possibility is higher drug pressure in the region. For example, household survey data from 2003 to 2015 suggests ACT use among symptomatic children was significantly higher in Eastern Africa compared to Central or West Africa, particularly around 2015 (Bennett et al., 2017). During this time in Uganda, 70.2% of symptomatic children received an ACT, compared to an average of 19.7% across the continent (Bennett et al., 2017). Inappropriate use of artemisinin monotherapies has also been observed in some areas of Uganda, with inadequate dosing of artemether-lumefantrine observed in ~12% of children and ~16% of adults in some districts (Wang et al., 2018). However, anecdotal reports also suggest this issue occurs more widely across Africa. Another possibility is that unstable transmission rates of parasites in East and Northeast Africa have promoted the development of ART-R, because when there is a switch from low to high malaria incidence in an area, host immunity is temporarily weakened and drugs need to be used more frequently (Rosenthal et al., 2024). This is relevant in East and Northeast Africa, as there are many highland regions which are known to be centres of unstable transmission (Rodó et al., 2021) and in particular may have been a factor in northern Uganda, where the discontinuation of an effective indoor residual spraying program could have contributed to a sudden resurgence in malaria cases and an increase in drug pressure (Conrad et al., 2023). Indeed, Uganda accounted for 5% of all global malaria cases in 2022, with higher transmission rates relative to many other African countries (only Nigeria and the DRC had more cases in that year; World Health Organization, 2023).

#### Could fitness costs, genetic backgrounds, or ecological factors slow the emergence of ART-R in Africa?

Although fitness costs of *kelch13* mutations are interesting conceptually, what potential do they have to slow the emergence of ART-R in Africa? Despite WHO validated/candidate *kelch13* mutations having arisen several times in East and Northeast Africa, so far there does not seem to be strong selective pressure for resistant parasites across the continent (Section ‘Rest of the World’; Rosenthal, 2021; Balikagala et al., 2021; Uwimana et al., 2020; Rosenthal et al., 2024). This suggests these mutants are being outcompeted in many areas, possibly due to a combination of higher fitness costs and different genetic backgrounds, alongside differences in competition between parasites (Cohen et al., 2012; Wasakul et al., 2023). For example, elevated transmission rates, combined with lower levels of drug pressure, may increase the disadvantage generated by fitness costs (Abebaw et al., 2022; Agaba et al., 2022; Bylicka-Szczepanowska and Korzeniewski, 2022). This is reflected in African countries where ART-R is relatively more common, such as Eritrea. In some cases, these are areas which have made substantial reductions in total malaria burden and reduced transmission, both of which may mean the fitness costs of resistance mutations are less important, giving resistant genotypes a larger advantage (Fola et al., 2023). Although local-context specific, this could slow the emergence and spread of ART-R in some areas of Africa.

However, it is important to note that the role of fitness costs and genetic backgrounds in determining population level parasite dynamics remains mostly speculative. To date, many of these effects have only been established *in vitro* (see Box 1 for details), and in *in vivo* settings, these costs likely interact with many other important factors which affect the relative competition between parasites, including drug use, vector dynamics, and antigenicity (Cohen et al., 2012; Myers-Hansen et al., 2020; He et al., 2024). Moreover, even if these factors do play a role, it is clearly still possible for ART-R to emerge independently on divergent genetic backgrounds and can be associated with varying degrees of selective pressure depending on local conditions, as seen in Southeast Asia (Takala-Harrison et al., 2015). Lastly, while the focus of this review has been on *kelch13* markers, it is not implausible that resistance in Africa could emerge through an alternative mechanism, and the fitness dynamics determining its spread may differ substantially (Demas et al., 2018; Ndiaye et al., 2021; Henriques et al., 2014).

In addition to parasite-specific factors, ecological and epidemiological differences between Africa and Southeast Asia could play a role in slowing the emergence of ART-R. For example, higher levels of population immunity among African populations can reduce parasite biomass during infection, lowering the likelihood of needing treatment and therefore decreasing drug pressure (Doolan et al., 2009). High rates of transmission and mixed-genotype infections can further increase within-host competition, reducing the likelihood that resistant parasites reach fixation. Moreover, the high genetic diversity generated by intensive transmission may have the effect of breaking up resistant haplotypes, hindering their spread (White et al., 2009). While the relative impact of these factors remains difficult to quantify, their combined effect may partially explain the slower rise of ART-R in Africa to date.

Future studies should work toward combining genomic surveillance of parasites with *in vitro* and population level estimates of effects on both transmission and fitness, as this may allow for improved modelling of epidemiological dynamics. This should be combined with whole-genome sequencing, as this would allow improved understanding of the role of these factors on ART-R dynamics. If their relative importance could be quantified, it could be crucial for genomic surveillance, as it could affect the relative priority given to tracking cases of specific mutations/genetic backgrounds and their spread between areas (Mayor et al., 2023).

### Rest of the world

**Figure 7.**
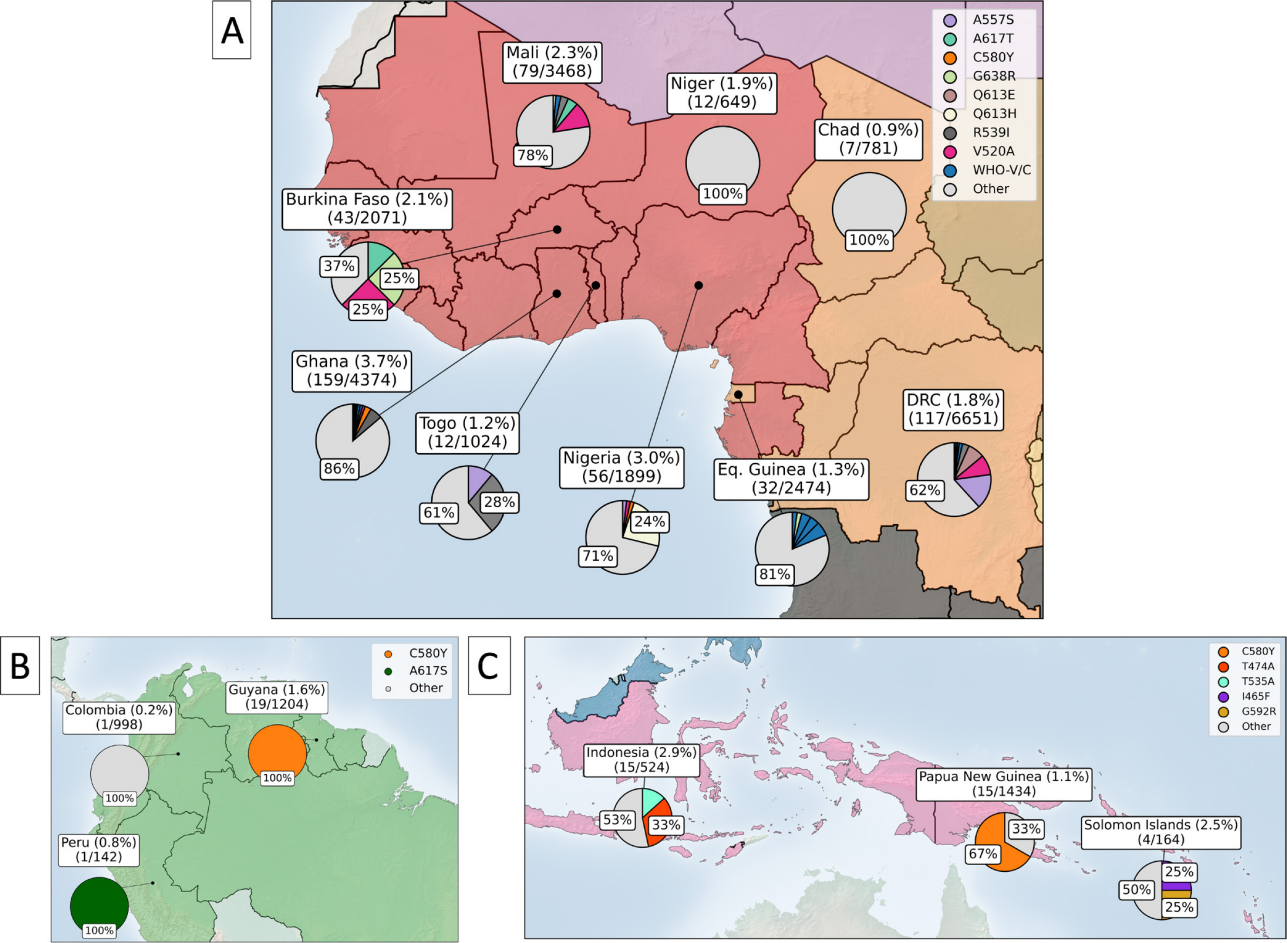
Distribution of *kelch13* propeller domain markers across West and Central Africa (**A**), South America (**B**) and Oceania (**C**). The percentage of samples with any *kelch13* mutation is included in the country labels, above the number of samples with any *kelch13* mutation, and the total number of samples collected for each country, respectively. Among only the samples with any observed *kelch13* propeller domain mutation, pie charts show the proportions with markers of interest, where proportions above 25% are labelled accordingly. ‘Other’ denotes several low-frequency mutations in the propeller domain which are not WHO validated/candidate markers, pooled into a single category.

We identified many *kelch13* mutations circulating across the rest of Africa, in particular West and Central Africa (Figure 7). For example, WHO validated/candidate mutations were detected at very low frequencies in Ghana, Mali, Nigeria, Equatorial Guinea, and the DRC (Figure 7). However, most mutations in West and Central Africa did not have WHO-validated/candidate status and therefore have unknown phenotypic effects. For instance, non-WHO validated/candidate *kelch13* mutations were detected in Benin and Burkina Faso in West Africa, and in the Central African Republic, Chad, the DRC, and Equatorial Guinea in Central Africa (Figure 7). While the phenotypic effects of these mutations are unclear, some may affect phenotypic resistance. For example, we observed a mutation, R539I, at the same *kelch13* position as the WHO-validated R539T in Togo, Ghana, Mali, and the DRC (Figure 7). This mutation should be surveyed closely and assessed in phenotypic assays of ART-R due to its position at a known resistance locus in *kelch13*.

WHO validated/candidate mutations were uncommon in other populations. In South America, one WHO mutation, C580Y, was detected in 19 samples from Guyana (Figure 7). Aside from this, only two other mutations were detected in South America, each in one sample (A617S in Peru in 2016 and A504T in Colombia in 2018; Figure 7). In Oceania, *kelch13* mutations were present in Indonesia, Papua New Guinea, the Solomon Islands, and Vanuatu. Evidence of ART-R was found in Papua New Guinea, where C580Y was present in 10 samples. The T474A was also circulating in Oceania, predominantly in Indonesia. From 1998 to 2020, 25 non-WHO validated or candidate mutations were detected in Oceania. A675V, F446I, R539T, and R561H were also present in India (Figure 5). These markers are also seen across Southeast Asia (Figures 5 and 6), highlighting the potential for ART-R in India. In contrast, in Far-East South Asia, non-validated/candidate mutations were detected in only five samples (0.002% of population). Similarly, Afghanistan, Iran, and Pakistan all showed very low proportions of mutant samples (<0.2% of samples), none of which had WHO validated/candidate status.

## Anticipating the next stage of ART-R in Africa

Given the emergence of ART-R in Africa, one obvious question is: what are the most likely scenarios for its spread? With the lessons learned from ART-R in Southeast Asia, it is useful to consider potential future scenarios on how ART-R might emerge and spread in Africa within the near future. Here, we articulate three possible scenarios regarding the frequency of *kelch13* mutations in Africa: a spread of similar magnitude to that of Southeast Asia, and two more optimistic scenarios. We then discuss broader trends we might observe going forwards, including the human cost of ART-R in Africa, the effect of mitigation efforts, regional variation in ART-R and its dynamics over the next few years. In doing so, we hope this will inform long-term strategic thinking on mitigation strategies, and in properly allocating resources for future surveillance. It is important to note that these scenarios are largely speculative and need to be continually updated with new data as the situation progresses.

### Scenario 1: Continued or increased spread of ART-R across Africa, similar to that seen in Southeast Asia

The emergence and spread of ART-R throughout Southeast Asia provides an example of how the situation might develop in Africa. In Southeast Asia, ART-R emerged in several independent locations, before a single drug-resistant lineage spread across the region (Hamilton et al., 2019; Imwong et al., 2017). The C580Y mutation then reached near-fixation (~86%) in parts of Cambodia within a period of around 12–16 years (Figure 5; Noedl et al., 2008; Dondorp et al., 2009; Kagoro et al., 2022). This offers one plausible scenario as to how ART-R might progress in Africa, although it does not preclude the possibility that it could spread even faster than in Southeast Asia. It should also be noted that a worse possible scenario would be the spread of ‘full’ (as opposed to ‘partial’) artemisinin resistance, but since this has not been reported in the field, a more likely scenario is the continued spread of ART-R.

**Figure 8.**
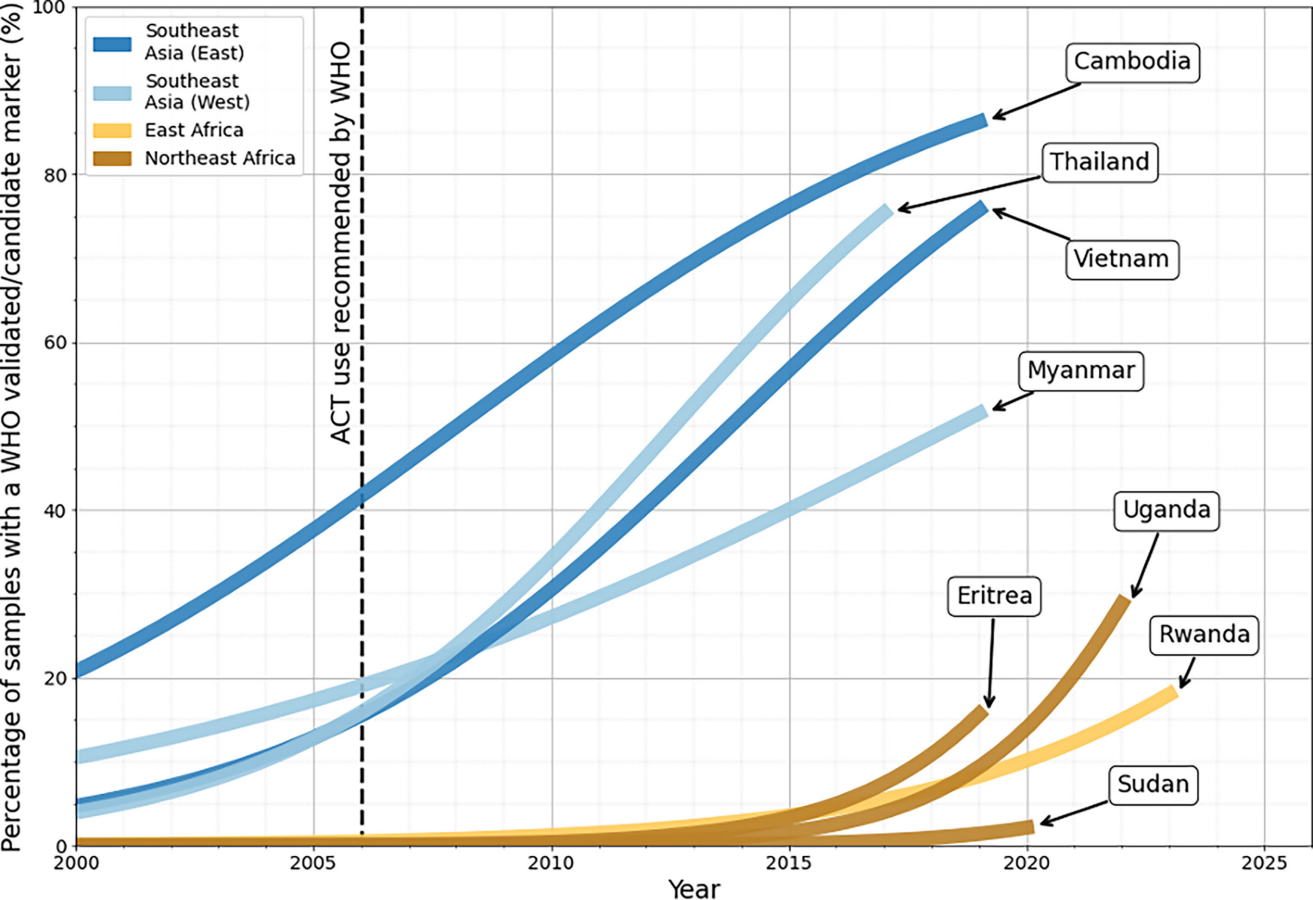
Proportion of samples with a WHO validated/candidate *kelch13* mutations over time (Table 2). Each country is shown using a logistic regression curve fitted to the observed data (see Figure 8—figure supplement 1 for model fits). Timepoints with no line indicate years with no available data. The black dashed line highlights 2006 - the year in which the WHO recommended ACT therapy as a first-line treatment measure in Africa (World Health Organization, 2006). Notably, the rapid increase in prevalence of WHO *kelch13* markers in East and Northeast African countries is similar to that observed in Southeast Asian countries between 2007 and 2016. However, it is also important to note these trends have so far only been observed in a few countries in East/Northeast Africa and are based on smaller sample sizes (Assefa et al., 2024). While these curves indicate the frequency of resistance has increased, we have deliberately avoided quantitative extrapolation beyond the most recent observed time range. This analysis should therefore not be interpreted as a predictive model.

As of 2024, this ‘partial’ resistance has now emerged independently in at least Uganda, Tanzania, Rwanda, Sudan, and Eritrea (Conrad et al., 2023; Uwimana et al., 2020; World Health Organization, 2022a). Importantly, there is evidence of an increase in the proportion of samples with mutant *kelch13* markers in several of these countries since 2014, with similar increases in frequency to those observed in Southeast Asia around 10–15 years ago (Figure 8). If these areas were to follow a similar trajectory to Cambodia, we could see frequencies of over 54% delayed clearance under ACT treatment by around 2030 (Slater et al., 2016). However, we stress that the trends presented here are illustrative rather than predictive and do not represent model-based projections (Figure 8). Additionally, the most recent data for Southeast Asia were from 2019, so these increases may not reflect more recent trajectories of ART-R in the region.

**Figure 8—figure supplement 1.**
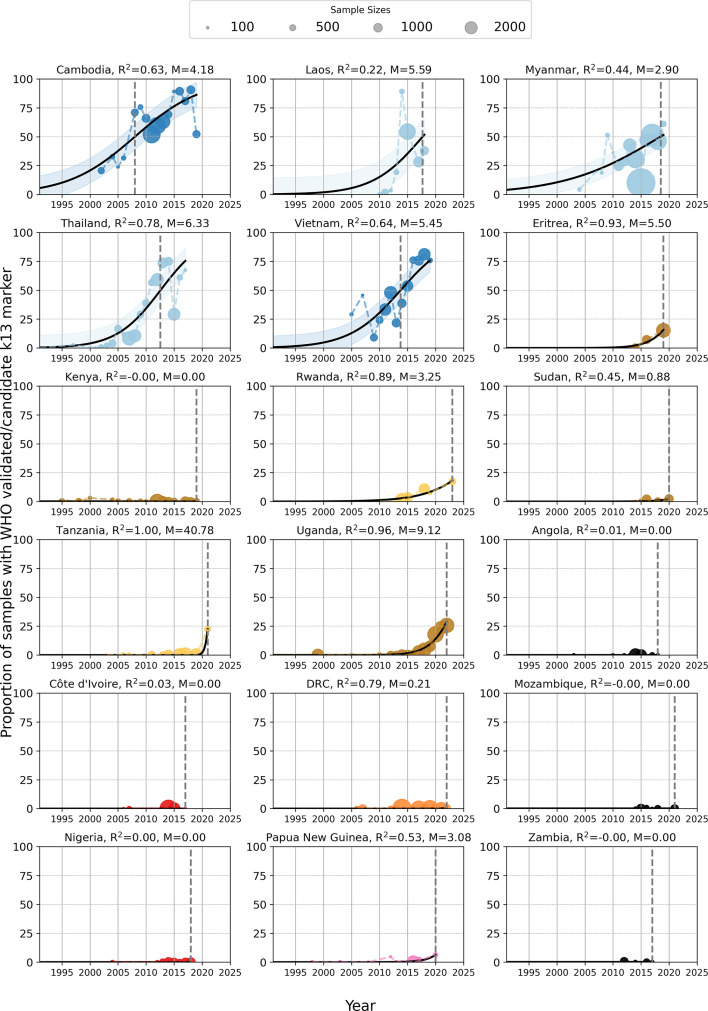
Percentage of samples with WHO validated/candidate *kelch13* propeller mutations over time. The observed data for each country is shown as a dashed line, coloured by population. The black line is the curve of a logistic model fitted to the observed data, which is forced to begin at 0 and end at 100%. The size of each point represents the number of samples collected in that country for each year. Time points with no line or value indicate years with no available data. Countries with fewer than 3 years with at least 25 samples each were excluded. Countries which showed no clear increase during this time were also excluded (Mali, Guyana, India, Ethiopia, China, and Equatorial Guinea). The grey dashed line highlights the year in which the fitted curve reached its steepest point - that is the year with the fastest increase in frequency. The goodness-of-fit of the logistic curves varied substantially across countries (R^2^=0.22–1.00). Notably, Tanzania had a very high growth rate, although this was based on a single year where the frequency was above 2%. To assess the robustness of the descriptive fits, we performed sensitivity analyses using bootstrap resampling within country-year groupings and a jackknife procedure where each individual year was excluded and the curve refit accomplished. These consistently reproduced the overall pattern of rising ART-R frequencies. While exact slopes and inflection points varied, the trend of increased ART-R prevalence in East and Northeast Africa remained robust. We emphasise that these are descriptive summaries of observed data and are not intended as predictive models.

Nevertheless, this scenario is further supported by estimates of selection coefficients in Uganda, which suggest current selective pressures are comparable to those observed in Southeast Asia between 2003 and 2018. These estimates imply the frequency of mutant *kelch13* parasites could reach as high as 95% between 2028 and 2033 (Meier-Scherling et al., 2024). Modelling also suggests that under the continued use of ACT therapies, *kelch13* mutations could become established in some areas (up to 57–88% frequency depending on model assumptions), although this will depend on their initial frequency and could be reduced using TACT strategies (see Box 2; Nguyen et al., 2023b).

Box 2. **What treatment strategies could slow the spread of treatment failure?**

ART-R is a major concern because of its broader role in ACT treatment failure. In essence, ART-R increases selective pressure for partner drug resistance, which in turn increases the likelihood of ACT treatment being unsuccessful. Several strategies have been proposed to slow the spread of treatment failure, including ACT cycling, multiple first-line therapies (MFTs), and triple artemisinin combination therapies (TACTs). Each of these methodshas been used at various times and locations, although all have varying costs, benefits, and degrees of evidence for their efficacy (Boni, 2022; van der Pluijm et al., 2021; Chen and Hsiang, 2022). It is important to note under each of these strategies, mutants which are resistant to both artemisinin derivatives and partner drugs may eventually arise (Box 2—figure 1). Having a strategy for if this occurs will be paramount, and will require careful genomic surveillance to identify cases where they emerge (Mayor et al., 2023).

*ACT cycling*

As of 2022, the WHO recommends a policy of ‘ACT cycling’, which involves using a single first-line ACT, before switching to a second once efficacy decreases below a certain threshold in the population (~ 90%) (World Health Organization, 2022a; World Health Organization, 2015a). Theoretically, this should reduce the frequency of resistance to the first drug, by providing time for resistance to decline in frequency while the second drug is in use (Box 2—figure 1; ; Nguyen et al., 2023a; Boni, 2022; Boni et al., 2008). This strategy initially proved effective in Cambodia, where artesunate-mefloquine was replaced with dihydroartemisinin–piperaquine, before reverting back after resistant parasites reduced in frequency (Rasmussen et al., 2022; Ross et al., 2018). However, there is some evidence to suggest the strategy later drove the emergence of triply resistant mutants in the northeast of the country (Rossi et al., 2017).

*Multiple front-line therapies*

More recently, several countries have adopted the use of MFTs, that is using multiple ACT therapies concurrently within a population (World Health Organization, 2021). Importantly, modelling studies have found MFTs have similar efficacy to cycling strategies but are less likely to accelerate the emergence and spread of resistance (Nguyen et al., 2015; Boni et al., 2008; Smith et al., 2010). This work suggests MFTs are most effective when using partner drugs with different mechanisms of action, as this reduces the chances of cross-resistance to both drugs (Li et al., 2024). However, the WHO has stopped short of recommending MFTs more generally (World Health Organization, 2013), citing contradictions in results from modelling work, and challenges in locations with both high drug use and resistance (Boni, 2022; Antao and Hastings, 2012). For example, the high frequency of the artemisini- resistant lineage in Cambodia means there is a lack of alternative treatments should MFTs fail (Rasmussen et al., 2022; Ross et al., 2018).

*Triple artemisinin-based combination therapies (TACTs)*

Another alternative is TACTs, which combine an artemisinin derivative with two partner drugs. Theoretically, this method preserves treatment efficacy, because of the rarity of parasites acquiring resistance to both partner drugs over a course of treatment (van der Pluijm et al., 2020). Recently, large-scale clinical trials in Southeast Asia have demonstrated TACTs are effective at national scales, even in situations where multidrug-resistant parasites are present (Peto et al., 2022; van der Pluijm et al., 2020). Modelling studies suggest that TACTs could significantly delay the emergence and spread of ART-R compared to other strategies (Zupko et al., 2023; Li et al., 2024; Nguyen et al., 2023a). However, their effectiveness diminishes once resistant parasites reach a threshold frequency in the population, which can be as low as 1%. A further drawback of TACTs is that because three drugs in total are being administered, the frequency of negative side-effects on patient health is more common (Peto et al., 2022; van der Pluijm et al., 2021; van der Pluijm et al., 2020). While promising, for TACTs to be useful at population scales, several practical issues need to be ironed out, such as correct dosing and formulation of partner drugs for overall safety, alongside increased cost-effectiveness and regional availability (for a review, see Nsanzabana, 2021). Moreover, their use requires public acceptance, as the reduced risk of emergent resistance is being balanced against an increased risk of side effects for any individual taking the drugs (van der Pluijm et al., 2021; van der Pluijm et al., 2020).

**Box 2—figure 1.**
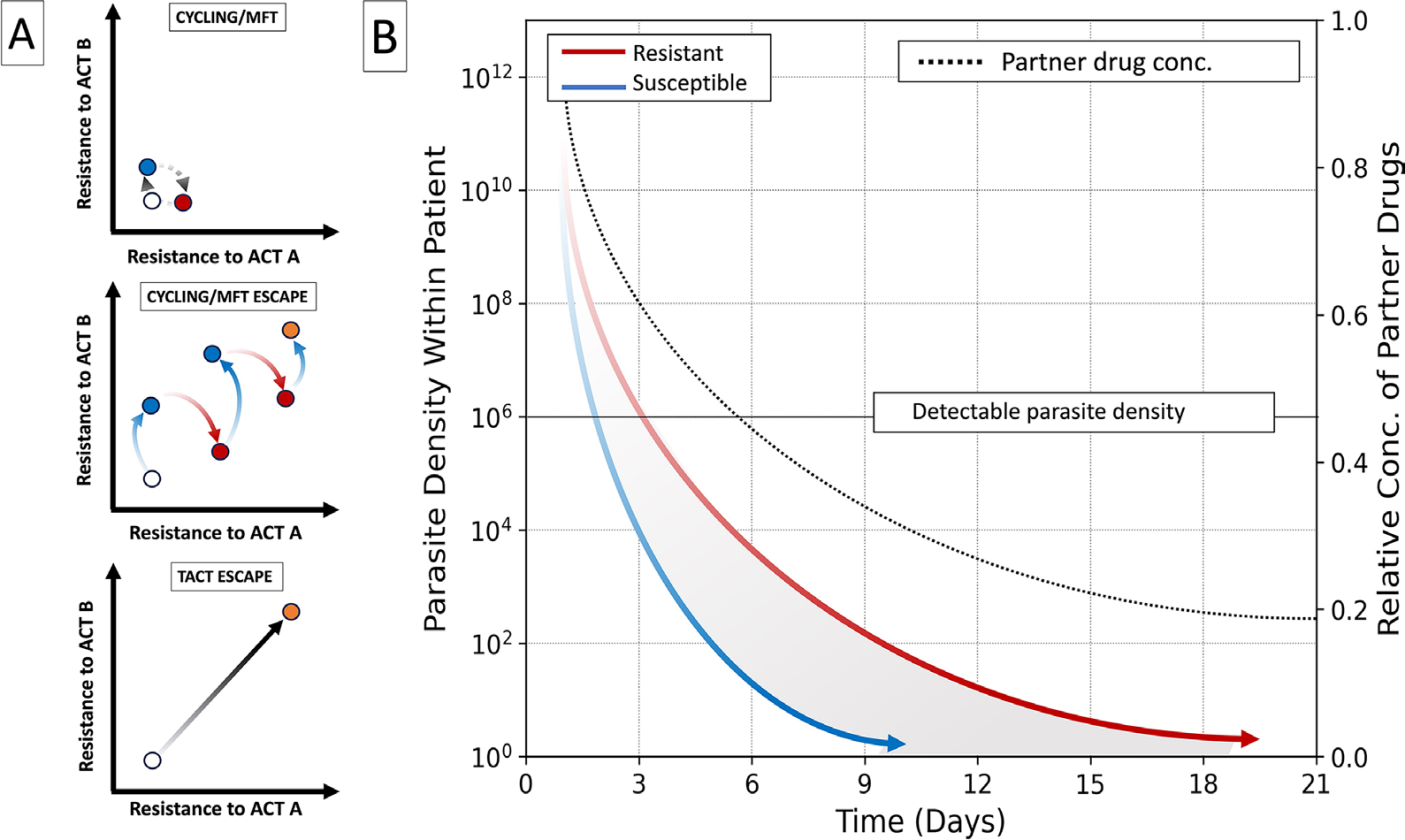
Evolutionary scenarios and within-host dynamics under cycling, MFT and TACT strategies. (**A**) There are three possible evolutionary scenarios during cycling, MFT or TACT strategies. In the idealised cycling/MFT scenario (top panel), resistance will cycle between two states of elevated resistance to a given ACT combination therapy, but not both, at any given time (red/blue points). However, these strategies could promote dual resistant escape mutants through stepwise increases over time (middle panel). This may occur even if resistance to any individual ACT decreases in the short term (red and blue lines). In contrast, triple artemisinin-based combination therapies (bottom panel) may directly select for triple resistant escape mutants (black solid line). While the likelihood of this occurring may be lower than under cycling or MFT strategies, this could result in mutants which are resistant to multiple partner drugs, leaving limited options to change strategy image adapted from Baym et al., 2016. (**B**) TACTs involve the use of two partner drugs throughout treatment. Here, a theoretical example of parasite density over time within a patient is shown for artemisinin-resistant and -susceptible parasites (red and blue lines respectively). In cases where parasites are resistant to artemisinin, their density remains higher after initial artemisinin exposure (~3 days), resulting in more parasites for the partner drug to treat (shaded area), and therefore stronger selection for resistance to that drug. TACTs use two partner drugs (black dashed line), which in theory means selection for any individual drug is lower, and the chance of triply resistant parasites emerging within any given infection is less likely Bushman et al., 2018 image adapted from Hanboonkunupakarn and White, 2022.

These increases in ART-R would in turn increase selective pressure for resistance to partner drugs, raising the likelihood of overall treatment failure. Indeed, there is evidence of treatment failure potentially occurring in some areas of Angola and the Democratic Republic of the Congo (Dimbu et al., 2024;Moriarty et al., 2021; Dimbu et al., 2021; World Health Organization, 2022a). Were such failures to become widespread, the human and economic consequences for the region would be disastrous (see Section ‘Widespread ART-R in Africa would be associated with significant human and economic costs’). Alarmingly, the observed increases in ART-R across East and Northeast Africa in recent years suggest this scenario is fast becoming a reality.

### Scenario 2: ART-R is limited in Africa by additional control interventions

It is clear that a massive and coordinated effort is urgently required to prevent the worst effects of ART-R from being realised (Dhorda et al., 2024). This will require several mitigation strategies to be implemented to reduce the emergence and spread of ART-R. These strategies include the wide-scale implementation of Triple Artemisinin Combination Therapies (TACT), or possibly Multiple Frontline Treatment (MFT) strategies (see Box 2), which modelling suggests could reduce treatment failure by 49–92% over a period of 5 years (Nguyen et al., 2015; Zupko et al., 2023; Li et al., 2024). If these strategies were implemented quickly and effectively, it may be possible to limit mutant *kelch13* to low frequencies in most areas, although regions where ART-R has already increased in frequency, such as Uganda, may see smaller reductions. These strategies could be combined with the recently approved RTS,S and R21/Matrix-M vaccines, which in addition to improved vector control and case management, could reduce transmission chains in regions of high ART-R (Manzoni et al., 2024; World Health Organization, 2022a; Datoo et al., 2024). This could provide much-needed time to develop additional antimalarial treatments. However, while these strategies will likely reduce overall malaria burden, it should be noted they may actually promote resistance in the short term (see Section ‘The drive towards malaria elimination may promote resistance in the short term’; Wasakul et al., 2023).

However, whether TACTs/MFTs could be implemented quickly and effectively is dependent upon overcoming a series of financial, systemic, and ethical challenges (Tindana et al., 2021; de Haan et al., 2023; Kokori et al., 2024). Administering and cycling multiple drugs will inevitably increase costs and place higher demands on procurement, storage, and distribution systems. Moreover, TACTs are not yet included in many national malaria treatment guidelines, and gaining support from global health authorities such as the WHO may be complex and time-consuming, yet is essential for wide-spread adoption. Lastly, there are also ethical considerations in asking patients to take treatments with additional potential side effects, but without immediate personal benefit, and raises concerns around adherence and acceptability. Their rollout will need to be supported by robust surveillance systems to guide deployment based on partner drug resistance and local ART-R dynamics, alongside strong operational capacity and community engagement (Straimer et al., 2022).

The success of these strategies may also depend on the speed with which they are implemented. For example, introducing TACTs after ACT-resistant parasites have reached even low frequency would likely have less of a preventative effect, with Nguyen et al., 2015 estimating this frequency could be as low as ~1%. Moreover, the size and scale of parasite populations in Africa means any interventions will be complex and time-consuming, giving ART-R mutations a chance to rise to high frequency before mitigations are in place (World Health Organization, 2022a). It is therefore a race against time to implement these strategies as quickly and effectively as possible, both to minimise the spread of ART-R and reduce overall malaria burden (Dhorda et al., 2024). Given progress in reducing malaria burden in Africa has mostly plateaued since 2015, any single strategy is unlikely to reduce incidence to a level where widespread ART-R would not have dire consequences (Dhorda et al., 2024). It will therefore be crucial to combine mitigation strategies with careful genomic surveillance of parasite populations, as it may be possible to minimise the short-term spread of ART-R by tailoring interventions to local conditions.

### Scenario 3: Lower frequency and slower spread of ART-R in Africa than Southeast Asia

While Scenario 1 and 2 are more likely scenarios, there are some reasons to think ART-R in Africa may not reach as high frequencies as Southeast Asia, or that if it were to, it may at least occur more slowly (Rasmussen et al., 2022). For example, some areas of Southeast Asia, such as Myanmar, showed a slightly lower overall frequency and slower spread of ART-R than Cambodia and Thailand, with several mutations reaching high frequency (Figure 5; Tun et al., 2015; Kagoro et al., 2022). One possibility is that differences in the phenotypic effects of *kelch13* mutations (e.g. clearance rate or fitness costs), or genetic backgrounds, slowed the spread of ART-R in these regions. Another possibility is that ecological factors have limited or slowed the spread of ART-R in Africa. For example, many African populations have higher rates of asymptomatic infection, stronger population immunity and greater parasite diversity than Southeast Asia (Wasakul et al., 2023; Abebaw et al., 2022; Agaba et al., 2022; Bylicka-Szczepanowska and Korzeniewski, 2022). These factors are likely to increase within-host competition, which may reduce the chance that resistant parasites increase to fixation (Bushman et al., 2018). Regional differences in vector ecology may also play a role. Variation in mosquito species, biting behaviour, or transmission patterns could influence how quickly resistant parasites are able to spread (Churcher et al., 2015; Sinka et al., 2010). While the role of these factors remains speculative, if they do play a role, their combined effects may be larger in African contexts, both because the effects of immunity and competition are more pronounced, and because the parasite population and geographical area in which resistance needs to spread are much larger, slowing its relative spread.

Differences in both drug policy and treatment quality may also affect the spread of resistance. For example, Southeast Asia has had more consistent drug policies, different first-line ACT choices, and higher-quality ACT distribution than areas of Africa, which may have exerted stronger selection pressures for resistance (World Health Organization, 2022a). Similarly, drug pressure may be lower in parts of Africa, because higher immunity reduces the rate of symptomatic infections, meaning they are less likely to require drug treatment (Doolan et al., 2009; Whitlock et al., 2021; Ataide et al., 2017). The presence of substandard and counterfeit drugs in Africa may mean there is also reduced drug use in these areas (Tun et al., 2018; Nguyen et al., 2023a). In East Africa, for example, the presence of counterfeit artemisinin-based drugs has been confirmed–one study in Uganda found ~37% of 57 stores were selling counterfeit ACTs (Björkman Nyqvist et al., 2022), and anecdotal reports exist of fake artesunate from Tanzania and Cameroon (Newton et al., 2006). The effect of these drugs likely depends on their contents: if counterfeit ACTs contain insufficient doses of artemisinin, this is likely to increase selection for ART-R. In contrast, if counterfeit ACTs containing no artemisinin are widespread in Africa, this may lessen selection for ART-R—although of course at the cost of effectively treating patients with malaria.

While it is difficult to estimate the precise effect these combined factors might have, one scenario could be ART-R frequencies in Africa will be similar to regions of Southeast Asia which have lower prevalence, such as Western and South Myanmar (Kagoro et al., 2022). In this case, we may regularly see moderate frequencies of resistant *kelch13* markers, but these could reach much higher frequencies in some regions, depending on the success of mitigation efforts (World Health Organization, 2022a). This would result in ART-R being relatively common, although it may not be the norm across the continent. Nevertheless, this would still put increased pressure on partner drugs, increasing selection for resistance and increasing the likelihood of treatment failure. These factors could also interact with mitigation strategies, which in some cases could negatively affect their rollout or overall effectiveness. It will be necessary to conduct careful genomic surveillance to estimate the effects of these trends on ART-R.

### Broader trends and likely trajectories of ART-R in Africa

#### Widespread ART-R in Africa would be associated with significant human and economic costs

Given limited alternatives to ACT treatment, any increase in ART-R which led to increased partner drug failure would be catastrophic for human mortality in the region (Conrad and Rosenthal, 2019). While the scale of the problem has been widely acknowledged, few studies have quantified the human and economic impacts of widespread ACT resistance (World Health Organization, 2022a; Slater et al., 2016; Lubell et al., 2014). Modelling work suggests highly negative outcomes, depending on the frequency of artemisinin/partner-drug resistance. For example, Lubell et al., 2014 found widespread ART-R (a conservative estimate of ~30% ACT failure rate) could cause an additional 116,000 deaths annually, with medical costs of US$32 million. Moreover, decreased productivity could be over US$385 million annually during the remaining lifetime of ACTs as the first-line treatment (Lubell et al., 2014). A second model considered several possible scenarios for ART-R and partner drug resistance in Africa, if resistance reached similar prevalence to various provinces in Southeast Asia (Slater et al., 2016). This model suggested if ART-R and partner drug resistance in Africa reached levels seen in Oddar Meanchey province in Cambodia (~54%), this could result in around 16 million additional cases per year (~7% overall increase) due to delayed parasite clearance and parasite recrudescence (Slater et al., 2016). Were this to take place, this could mean an additional 20,000 deaths and an economic impact of US$1.1 billion per year due to increased morbidity and mortality (World Health Organization, 2022a; Slater et al., 2016; Hailu et al., 2017). Unfortunately, these models offer conservative estimates and may even underestimate the true severity of the situation. The impact could be significantly worse if additional factors, such as diminished economic growth or lower educational outcomes, were taken into account (World Health Organization, 2022a; Slater et al., 2016; Lubell et al., 2014).

#### The drive towards malaria elimination may promote resistance in the short term

Given the potential scale of the problem, national and international public health agencies have developed extensive plans to reduce total malaria burden (World Health Organization, 2021; World Health Organization, 2022a). For example, the WHO has a stated goal to eliminate malaria in 35 countries by 2030 (World Health Organization, 2021; World Health Organization, 2022a). While their success so far has been debated (World Health Organization, 2022a; Rosenthal et al., 2019), as the total number of cases decreases, it is likely the proportion of drug-resistant parasites will increase (White, 2004; Scott et al., 2018). This is for two reasons; firstly, since ACTs are a main strategy in treating infection, and ACT use selects for resistant parasites, resistant parasites are more likely to increase in the population (White, 2004). Secondly, as the overall parasite population and rate of infection are reduced, this will decrease immunity within the human population (Agaba et al., 2022). As this occurs, the rate of asymptomatic infections–currently the most common kind of infection in many parts of Africa–may decrease, meaning more need for drug treatment and a further increase in selection for resistance (Bylicka-Szczepanowska and Korzeniewski, 2022).

This process is already being observed in some countries in Africa, such as Eritrea (Fola et al., 2023; Abebaw et al., 2022; Mihreteab et al., 2023; Jeang et al., 2024). Over the past two decades, concerted public health efforts, such as vector control, ACT treatment, and case management, have reduced infections in this region. However, some evidence suggests the success of these strategies means the proportion of ART-R parasites has increased, despite a reduction in overall burden (Fola et al., 2023). This trend has also been observed in several parts of Southeast Asia, such as Myanmar (Manzoni et al., 2024; Imwong et al., 2017; Imwong et al., 2020). This underscores the importance of careful genomic surveillance, as it can be used to identify patterns of selection on resistant parasites (Meier-Scherling et al., 2024). This would, in turn, inform mitigation strategies and help ensure that as overall parasite numbers are reduced, they are not replaced by resistant parasites.

#### The prevalence of ART-R is likely to vary substantially between regions

A consequence of variation in reducing malaria burden is that the frequency of ART-R is also likely to be highly variable across the African continent, particularly over the next 1–5 years. This is not only because different African countries currently have very different malaria burdens, but are likely to have different rates of success in eliminating the disease (World Health Organization, 2022a). This variable distribution is observed in Southeast Asia, where some regions, such as districts of Cambodia, show near fixation of mutant *kelch13* haplotypes, while others, such as West and South Myanmar, have less than 20% prevalence (Kagoro et al., 2022). Unfortunately, the consequence of this is that some regions could become hubs for the spread of ART-R into other areas (Ashley et al., 2014; Tun et al., 2017). A similar effect was also observed in other regions of Southeast Asia, where waves of selection assisted in spreading resistant lineages across the region (Ashley et al., 2014; World Health Organization, 2016; Tun et al., 2017). There are concerns these patterns could re-occur among African countries, as resistance-associated *kelch13* markers have so far only been observed in specific regions, such as Uganda, Rwanda, and Eritrea (Conrad et al., 2023; Uwimana et al., 2020; Agaba et al., 2022). So far, it is not possible to determine whether a clonal spread of resistant genotypes is spreading in all of these countries due to the limited number of samples, although evidence suggests that so far these instances have emerged independently (Conrad et al., 2023; Uwimana et al., 2020; Abdel Hamid et al., 2023; Agaba et al., 2022). This again highlights the importance of genomic surveillance and rapid data sharing, as when migration routes do arise, these can only be identified by monitoring and sequencing parasite populations, and used to inform mitigation strategies (Tun et al., 2017).

#### The dynamics we observe in the next few years will determine the prevalence of ART-R in 10 years’ time

The patterns of resistance we see over the next 1–5 years will be crucial in determining the patterns we observe over the next decade. This is for four main reasons; firstly, the areas in which resistance first emerges indicate areas where resistance is currently under strong selection and therefore likely to reach higher frequencies. This effect was observed in Southeast Asia, where the regions in which resistant parasites initially emerged gave a strong indication as to where they would later reach high frequency, such as Cambodia and Thailand (Kagoro et al., 2022; Imwong et al., 2017). Secondly, resistance frequencies may reach critical thresholds in some areas within the next 1–5 years, beyond which mitigation strategies will be more difficult. For example, modelling studies suggest MFTs and TACTs are less likely to be effective if they are implemented only when the initial frequency of resistance is already high, with efficacy decreasing substantially when this frequency exceeds 1%. Thirdly, these years will give a strong indication whether fitness costs and genetic background have a slowing effect on the spread of resistance in Africa, and this could assist in making predictive models for further spread. Lastly, we may see the initial effects of RTS,S and R21/Matrix-M vaccines during the next 1–5 years, and we can observe how this might affect selective pressures on the parasite (Datoo et al., 2024).

Unfortunately, this period is also the time in which it is most difficult to alter mitigation strategies, simply because their planning and implementation takes time and resources from public health organisations. The next 1–5 years will therefore be where genomic surveillance and rapid data sharing is most crucial, as this could provide a warning system to identify at-risk areas and provide indications on how to best mitigate the spread of resistance. Incorporating whole genome sequencing into routine sampling will be particularly important, as this would enable discovery of new markers of resistance and detection of signatures of genomic adaptation to antimalarials, such as clonal population expansion. Whole genomes would also offer improved inferences on parasite dynamics, such as migration, population structure, and selection pressure, all of which will be crucial in tracking the spread of resistance.

## The role of genomic surveillance going forward

Throughout this review, we have highlighted the vital role genomic surveillance has played in understanding the global emergence and spread of ART-R in *P. falciparum*. In Southeast Asia, genomic surveillance helped to identify instances of migration of resistant parasites between countries and quantify the frequencies of resistance in different areas (see Section ‘Spread of Artemisinin Partial Resistance’). Now, in East and Northeast Africa, genomic surveillance continues to demonstrate its usefulness, by identifying the independent emergence and spread of *kelch13* mutations in Sudan, Uganda, Eritrea, and Rwanda (Conrad et al., 2023; Uwimana et al., 2020; Mihreteab et al., 2023; Mohamed et al., 2020; Owoloye et al., 2021; Jalei et al., 2023). These discoveries were only possible due to the enhanced sensitivity of genomic surveillance in detecting low-frequency genetic variants - as several TES from the region suggested treatment efficacy remained high (>95%) (Assefa et al., 2024). However, TES primarily aids in understanding treatment failure due to partner drug resistance, so is not expected to inform on ART-R in the same way as genomic surveillance is able to do. Moving forward, it is essential to use the information gathered from both genomic surveillance and TES, along with lessons learned from ART-R in South-East Asia, to develop and implement mitigation strategies, such as properly allocating healthcare resources (Assefa et al., 2024; World Health Organization, 2015b; World Health Organization, 2021).

### The need for a more systematic approach to genomic surveillance

Genomic surveillance of ART-R in Africa is at a crucial inflection point. As of 2024, limitations on resources, time, and training, alongside the scale of parasite populations in Africa, mean surveillance efforts remain localised, often with limited numbers of samples from key areas (World Health Organization, 2022a; Nsanzabana, 2021). This is a problem because surveillance is most valuable as a warning system when it is detailed enough to identify low-frequency variants (e.g. less than 1%), and these can easily be missed if not enough samples have been analysed (Uwimana et al., 2020; Mayor et al., 2023; Asua et al., 2019; Boni, 2022). This highlights a fundamental challenge for the surveillance of ART-R, in that if resistance is circulating at lower frequencies, it is simply harder to detect using any method, and quantifying changes in those frequencies can be difficult without detailed sampling over time within the same areas (Mayor et al., 2023). While identifying resistance mutations at frequencies of around 5% or more is still very valuable, detecting mutations at lower frequencies gives more time to delay the establishment of resistant parasites or reduce their spread to neighbouring regions. These issues mean increases in resistance mutation frequencies may only be identified when they have effects on clinical efficacy (Zupko et al., 2023).

A significant increase in genomic surveillance is needed, alongside a more systematic approach to sample collection. Surveillance could be improved by using networks of health facilities which routinely monitor ART-R frequencies within regions and conduct longitudinal sampling of areas over time (Nsanzabana et al., 2018; World Health Organization, 2009; Mayor et al., 2023; Guillot et al., 2022). Adapting sampling for more novel surveillance approaches, including genomic surveillance, could help to maximise the available information on resistant allele frequencies and improve the detection of low-frequency variants (Mayor et al., 2023). This could involve performing a priori power analyses to guide sampling efforts, based on predefined estimates of mutation prevalence and desired margins of error (Mayor et al., 2023).

More generally, the complementary use of whole genome sequencing and amplicon sequencing will be crucial to best tackle ART-R. Both technologies are more sensitive for detecting low-frequency variants than traditional approaches such as sequencing using capillary electrophoresis. This is particularly important for molecular surveillance in Africa, where high proportions of mixed infections can hinder the identification of low-frequency variants. As amplicon sequencing is likely to continue to be the most accessible genomic sequencing method in the near future, we must strive to use whole genome information to inform and improve amplicon sequencing. For example, whole genome sequencing can shed light on the ‘unknown unknowns’ of ART-R, such as novel drug resistance mechanisms, including the effect of mutations in the N-terminal region of the *kelch13* gene, as well as those outside the *kelch13* gene, such as *pfcoronin*, *ubp1* and *pfap2mu*, which could then be incorporated into amplicon sequencing panels (Cerqueira et al., 2017). Aside from improving amplicon sequencing, whole genome data can also provide insights to partner drug resistance genotypes and parasite population dynamics (Amato et al., 2018; Parobek et al., 2017). Such information could inform models which characterise selective pressures in response to interventions like drug usage (Nguyen et al., 2023b). Whole genome data can also highlight important changes in genomic architecture which can be indicative of evolution in response to control measures (Miotto et al., 2015). For example, identifying copy number variations in drug resistance genes, or deletions and breakpoints which lead to rapid diagnostic test failures (Rocamora and Winzeler, 2020). Moreover, increasing overall whole genome sequencing data and making it publicly available as soon as possible after collection would ensure maximum value from surveillance efforts.

Lastly, a major focus should be on building sequencing and data processing capabilities in malaria endemic countries, particularly in the African continent. Currently, the substantive cost of reagents and issues with supply chain logistics can hinder efforts for in-country data generation (Neafsey et al., 2021; Hamilton et al., 2023). Additionally, training of local scientists in specialist fields such as bioinformatics, genomics, and data science would further enable the ability to turn sequencing data into interpretable findings (Ishengoma et al., 2019). Challenges exist around obtaining sustained funding for long and short-term data storage, while the difficulties of data compartmentalisation, standardisation, and administrative burdens will also need to be addressed to best leverage ongoing genomic surveillance efforts. Moving forwards, it will be critical to expand surveillance by enabling the use of sequencing more widely in Africa (Ishengoma et al., 2019). These improvements could include nanopore long-read technology, which has been demonstrated to be well-suited to surveillance needs through its increased portability, lower up-front costs, and real-time data output (Hamilton et al., 2023). Several combined strategies could vastly enhance the region’s ability to respond to future outbreaks, both for *Plasmodium* and other pathogens. These strategies include the systematic investment in local surveillance infrastructure and workflows, changes in sampling strategy (towards a framework informed by genomic surveillance), and a focus on building a critical mass of expertise in areas where gaps have been identified.

### Improved models to analyse large spatiotemporal datasets

In addition to enhanced genomic surveillance, developing new models to analyse increasingly large spatiotemporal datasets is crucial. Improved epidemiological models could characterise outbreaks as they unfold or even forecast how they may progress. For example, models which can estimate identity-by-descent among large numbers of parasites would enable inferences on population scale changes in genetic diversity (Miotto et al., 2013; Henden et al., 2018; Amambua-Ngwa et al., 2019). This capability would enhance quantification of selective pressures for ART-R, identify migration pathways between regions, and help categorise population diversity (Amato et al., 2018; Meier-Scherling et al., 2024; Amambua-Ngwa et al., 2019). Improved models could also study changes in the frequency of drug-resistant lineages and facilitate the interpretation of these changes in relation to other factors, such as drug use, vector populations, or climate change (Datoo et al., 2024; Ryan et al., 2020; Sherrard-Smith et al., 2022; Redding et al., 2024). Moreover, developing methods to identify novel resistance markers is crucial—not only for *kelch13* and partner drug resistance genes, but also for other genomic loci that may contribute to ART-R through complementary or polygenic mechanisms (Demas et al., 2018; Miotto et al., 2015; Nayak et al., 2024). For example, linkage disequilibrium has been identified between non-propeller mutations R255K, K189N, and K189T in *kelch13*, as well as between these mutations and other drug resistance linked genes such as *mdr1* in Mali (Coulibaly et al., 2022). While not validated or candidate mutations, K189T and R255K were recently associated with delayed parasite clearance in one study from Nigeria (Amusan et al., 2025). Initially, the search for novel resistance mechanisms could include polymorphisms in the N-terminal region of the *kelch13* protein, but should then extend to those outside the *kelch13* gene. This is especially important in African parasite populations, where resistance-conferring mutations beyond *kelch13* have already been identified and may interact with genetic backgrounds in ways that influence their emergence and spread (Miotto et al., 2015; Demas et al., 2018; Tumwebaze et al., 2022). This will be particularly important during the rollout of MFT or TACT strategies, as any triply resistant mutants which arise can be more easily identified using genomic surveillance, and this information could inform optimal timing to rotate drugs (Zupko et al., 2023; Kokori et al., 2024; Boni, 2022).

### Improved data sharing, public access to data and training opportunities

Ensuring that surveillance data and analysis methods are publicly available is crucial for the malaria community, as these resources are essential in building skills and capacity for bioinformatic analysis. Public access to large surveillance datasets has repeatedly empowered novel analyses on the basic biology of *Plasmodium*, possible only because of the open nature of these datasets and the willingness of the community to share data (Abdel Hamid et al., 2023). For example, recent analysis of the Pf7 open dataset of whole genome sequencing samples led to the identification of a novel ‘cryptotype’ of *P. falciparum*, present in 13 countries across Africa (Miotto et al., 2024). Additionally, making the outputs of genomic surveillance data available through centralised platforms like MalariaGEN, WWARN, and WHO’s malaria threat maps has provided the malaria community with invaluable resources to study the distribution, migration and spread of ART-R (Abdel Hamid et al., 2023; Sibley et al., 2007). Similarly, web applications like Pf-HaploAtlas have been developed to leverage these resources, facilitating analysis of genomic surveillance data without the need for specialised technical knowledge (Lee et al., 2024). It is imperative to continue this work by ensuring these tools and resources are accessible and user-friendly, particularly for those with limited training, and to support the malaria community in making their data publicly available as soon as possible after collection. This approach will enhance contributions and coordination within the community, and help in informing strategies to mitigate the spread of ART-R.

## Conclusions and outlook

Given the emergence of ART-R associated with *kelch13* markers in Africa, it is likely only a matter of time before these begin to spread widely across the continent (Dhorda et al., 2024; Rosenthal et al., 2024). This review has highlighted the crucial role of genomic surveillance in alerting public health agencies to the spread of ART-R and in planning the use of limited resources to contain it. Recent years have seen limitations on funding, political instability, and other public health priorities, such as the COVID-19 pandemic, divert already limited resources away from genomic surveillance of *P. falciparum*, jeopardising the surveillance of ART-R in Africa (Lyimo et al., 2022; Mensah et al., 2022). Despite these challenges, it will be crucial to continually develop and champion the use of genomic surveillance as a vital part of the surveillance toolkit for ART-R, which also contains methods such as treatment efficacy studies and *in vitro* phenotypic studies. Without coordinating these surveillance tools, increases in ART-R may only be identified when they have already spread widely, negatively affecting patient outcomes and compromising our ability to implement mitigation strategies (Zupko et al., 2023). This means continuing surveillance is vital, not only for containing the spread of ART-R, but also for minimising the human costs it will cause.

## Methods

### Data aggregation

We compiled publicly available *kelch13* mutation data for 112,933 samples collected between 1980 and 2023, across 73 countries (Supplementary file 2, Source Data S1). This dataset, along with associated metadata, was sourced from the MalariaGEN Pf7 release (Abdel Hamid et al., 2023), the Worldwide Antimalarial Resistance Network (WWARN) Artemisinin Resistance Molecular Surveyor, and our own literature search. For the purposes of this study, all BTB/POZ and propeller mutations (codons 349–726) are collectively referred to as ‘propeller mutations’. Mutations were classified according to their status for association with ART-R from the latest WHO guidelines - either ‘validated’, ‘candidate’, or ‘no status’ (Supplementary file 1). Parasites with the 3D7 reference *kelch13* sequence, or the mutation A578S, were considered susceptible to artemisinin (World Health Organization, 2022b). As only non-synonymous mutations were included in this study, samples with synonymous amino acid mutations only were classified as having the 3D7 reference sequence.

While we focused our analysis on the BTB/POZ and propeller domains, which include all WHO-validated and candidate ART-R mutations, additional variation exists in the N-terminal region of *kelch13*. Some of these upstream mutations may have functional or compensatory effects, although their role remains unclear. However, the WWARN dataset—which makes up most of our aggregated data—only includes sequences from codon 440 onwards, limiting our ability to systematically assess upstream diversity. To ensure consistency and comparability across sources, we therefore restricted analysis to the BTB/POZ and propeller domains. Samples with a mutation outside of codons 349–726 were therefore classified as having the 3D7 reference sequence. In some cases, samples with two *kelch13* mutations were identified. When one mutation was outside of the propeller domain, we counted only the propeller domain mutation for that sample. Where both mutations were detected within the propeller domain, we recorded both mutations for the sample, meaning it could be a mixed strain or poly-mutant.

Mutations were recorded alongside sample ID, continent, country, collection year, publication source, and method of SNP detection (Source Data S1 and S2). No exclusions were applied based on geographical or temporal metadata. Samples from each country were assigned to one of the 13 broader populations: South America, West Africa, Central Africa, North Africa, Northeast Africa, East Africa, Southern Africa, Western Asia, Eastern South Asia, Far-Eastern South Asia, Western Southeast Asia, Eastern Southeast Asia, and Oceania (Supplementary file 2), in accordance with the Pf7 dataset (Abdel Hamid et al., 2023). In Pf7, Kenya, India, and Thailand were split across two populations. We therefore assigned samples from these countries to the neighbouring population which overall held most samples. All samples from Thailand were allocated to Western Southeast Asia, all samples from Kenya were allocated to East Africa, and all samples from India were allocated to Eastern South Asia. Three extra populations were created for this analysis to account for samples which did not fit into pre-existing Pf7 populations: Southern Africa (Angola, Mozambique, South Africa, Zambia, Zimbabwe), North Africa (Algeria, Libya), Western Asia (Afghanistan, Iran, Pakistan) (Supplementary file 2). Pf7 samples from Mozambique were also re-allocated to Southern Africa.

In analysing and visualising these data, we applied a multi-level aggregation approach: while we provide the dataset at the sample level, our analysis and visualisations aggregate prevalences either at country, subpopulation or continent levels. This structure enables broader geographic insights and facilitates interpretation of large-scale trends. Of the two *kelch13* molecular surveyor databases, the WHO Threats Map presents figures at the district level (World Health Organization, Global Malaria Programme, 2024), and WWARN provides both data and visualisations at the study level. Our decision to use country- and region-level aggregation was driven by the need for comparability across heterogeneous data sources and to maximise sample sizes. This approach is particularly suited for identifying geographic and temporal patterns at a global scale, whereas study-level and site-specific surveillance—such as that offered by WWARN and WHO Threats Map—is useful for ongoing monitoring efforts and intervention planning.

### Source-specific data processing

#### Pf7

Whole genome data from 16,203 QC-passed Pf7 samples were processed using custom Python scripts to generate haplotypes for *kelch13*. During QC, duplicate samples were removed. For each sample, we applied nucleotide variants from the GT field of the sample’s VCF file to the 3D7 reference sequence. These sequences were translated into amino acids, and mutations were identified. We excluded any sample with a missing genotype call (n=1,056). After removal of heterozygous samples (n=2,292), 12,855 samples from Pf7 were included in the final dataset.

#### WWARN database

*A*ll *kelch13* data from WWARN’s Artemisinin Molecular Surveyor were downloaded. We reviewed 63 studies where only a range of years, rather than a single year, was provided. If we were not able to gather single-year data for all samples from the original publication, we recorded the median year of the range for the study. In cases where the year range was 2 years, we selected the year with the largest number of samples. We investigated potential duplicate or missing data in the full texts of 34 WWARN publications that had different total numbers of recorded mutations and tested samples. Of these, 17 studies were included after correcting a mismatch between the publication and the WWARN data file and one was included after being flagged as containing multiple propeller mutations per sample. The remainder were excluded for containing heterozygous samples, or due to missing data for most samples, or because the publication was inaccessible. We assumed no other duplicate samples were included within the WWARN dataset. Ultimately, we included data from 291 WWARN publications covering 86,870 samples.

#### Literature search

To ensure our database was as comprehensive and up to date as possible, we performed an additional literature search for relevant papers published after 2020 to supplement the WWARN database. We searched the Web of Science and PubMed using: ‘K13 OR kelch OR kelch13 OR pfkelch13 AND (half-life OR parasite clearance OR resistance)’, restricting results to articles only. We also searched ‘kelch13 mutations *falciparum* artemisinin’ in Google Scholar for articles published between 2023 and 2024. We excluded articles which genotyped *kelch13* after ACT treatment (i.e. not day 0 patients) or in cultured lab strains, articles which did not provide country-level information or sample size information, articles which only provided genotypes for a subset of samples tested, articles already included in the WWARN database, and articles not available in English or not accessible. We yielded 28 additional unique studies after reviewing full texts and applying both the article and mutation inclusion criteria. We ensured that no missing data were present by checking that the total number of ‘tested’ samples and the number of samples with a reported *kelch13* genotype were equal. In any instance where wildtype genotypes were not reported and the number of samples with a *kelch13* mutation was below the total number of tested samples, we added wildtype counts to complete the study data. A further 13,208 samples were collected from this search.

## Additional files

Supplementary file 1. this should reduce the frequency of resistance to the first drCriteria for classifying *kelch13* mutations as validated or candidate markers of artemisinin partial resistance, based on WHO guidelines (World Health Organization, 2022b). Includes the current list of accepted mutations in each category.

Supplementary file 2. Populations included in this analysis, with countries and sample sizes listed for each. Ten populations are based on MalariaGEN’s Pf7 geographic/genetic groupings; three additional populations (Southern Africa, North Africa, Western Asia) were defined for samples not fitting existing groupings. “Proportion of population” = percent of samples from each country within a population. “Proportion of total” = percent of total dataset contributed by each country. DRC = Democratic Republic of the Congo.

Supplementary file 3. Number of unique *kelch13* propeller mutations identified in each population. C/V = WHO-classified candidate or validated marker.

Supplementary file 4. Proportion of total samples per year from each population.

Supplementary file 5. Summary statistics from logistic regression models of ART-R transmission dynamics in selected countries.

Supplementary file 6. Therapeutic efficacy studies (TES) in Africa reporting delayed parasite clearance or >10% 1455 treatment failure following ACT administration.

Supplementary file 7. Adoption of first-line ACT regimens by country and population as of end-2022, according to 1459 WHO reports. The number of countries in each population using each regimen for 1460 uncomplicated malaria. Abbreviations: A – artemether, L – lumefantrine, AS – artesunate, 1461 AQ – amodiaquine, MQ – mefloquine, DHA – dihydroartemisinin, PYR – pyronaridine, CQ – 1462 chloroquine, PPQ – piperaquine, PQ – primaquine.

Supplementary file 8. README file for Source Data S1.

Supplementary file 9. README file for Source Data S2.
